# Activating peptides for cellular uptake *via* polymerization into high density brushes†
†Electronic supplementary information (ESI) available: Full experimental details and additional figures are provided. See DOI: 10.1039/c5sc03417e



**DOI:** 10.1039/c5sc03417e

**Published:** 2015-11-10

**Authors:** Angela P. Blum, Jacquelin K. Kammeyer, Nathan C. Gianneschi

**Affiliations:** a Department of Chemistry & Biochemistry , University of California-San Diego , La Jolla , California 92093 , USA . Email: ngianneschi@ucsd.edu

## Abstract

The utility of peptide therapeutics is thwarted by an inability to enter cells, preventing access to crucial intracellular targets. Herein, we describe a simple and potentially widely applicable solution involving the polymerization of a minimally modified amino acid sequence into a high density brush polymer. Specifically, non-cell penetrating peptides can be rendered competent for cell entry by first including a single Arg or Lys in their amino acid sequence, if one is not already present, along with a norbornenyl unit. This modified monomer is then polymerized by ring opening metathesis polymerization (ROMP). To demonstrate the utility of this strategy, a known therapeutic peptide, which does not penetrate cells on its own, was polymerized. The resulting polymer proficiently entered cells while maintaining its intracellular function. We anticipate that this methodology will find broad use in medicine, increasing or enabling the *in vivo* efficacy of promising peptide therapeutics.

## Abstract 2

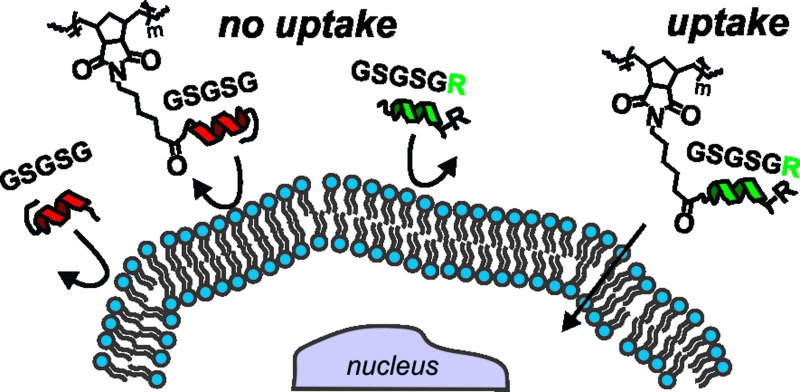
The utility of peptide therapeutics is thwarted by an inability to enter cells, preventing access to crucial intracellular targets.

## Introduction

The chemical diversity inherent to natural and unnatural amino acids enables the formulation of peptides that are selectively and precisely coded for interaction with target receptors and other biological surfaces. This ability has fostered the development and identification of unique natural, semi-synthetic and synthetic peptide sequences capable of diverse medicinal^1–4^ and diagnostic^5^ applications. Despite their promise, the clinical efficacy of many peptide-based therapeutic and diagnostic agents is severely hampered by three key obstacles: errant proteolysis, inefficiencies in cellular uptake, and size-dependent renal clearance.^1–4^ We recently described a strategy for protecting peptides from proteolysis in which peptides are packaged as high density brush polymers *via* graft-through ring opening metathesis polymerization (ROMP) of peptide-based monomers, generating structures that are resistant to proteolytic degradation.^6^ This strategy does not require chemical modification of the primary amino acid sequence and is, therefore, a facile approach to access formulations of protease-resistant peptides that maintain their inherent function. Here we demonstrate that, when polymerized into a high density brush polymer, peptides bearing at least one Arg or Lys can efficiently penetrate cells.

The biological target of most therapeutic agents resides in the cytosol or nuclei of cells. Therefore, potential therapeutic peptides that cannot gain entry into the interior of a cell are generally ineffective. Conventional strategies for conferring cellular uptake typically involve appending the peptide of interest to a cell penetrating peptide (CPP).^7^ CPPs, such as Tat and Arg8, are most often highly charged sequences that contain multiple copies of arginine (Arg). CPPs of this type have been shown to deliver a wide variety of conjugated cargo into cells. However, materials linked to CPPs in a linear arrangement maintain their susceptibility to proteolytic digestion.^8^ Thus, the development of general strategies that provide the needed dual function of protecting peptides from proteolysis while facilitating cellular entry have the potential to change the way peptides are prepared and delivered.

**Fig. 1:**
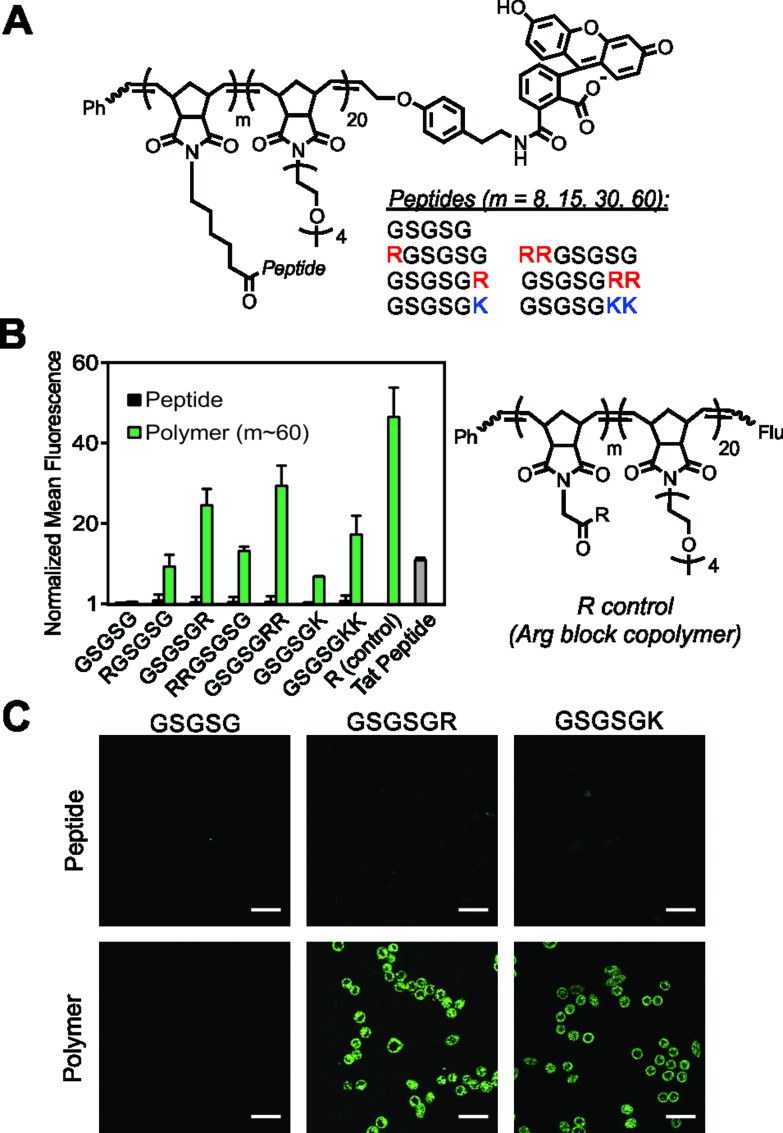
Cellular internalization of GSGSG polymers and analogues. (A) Chemical structure of peptide block copolymers. (B) Flow cytometry data showing fluorescent signatures of HeLa cells treated with the polymers (*m* ∼ 60) and their monomeric counterparts. All data are normalized to the vehicle (DPBS), which is assigned a value of 1. The R control is a block copolymer that contains a single Arg attached *via* a short linker to each polymer side chain of this first polymer block (*m* ∼ 60). “Flu” is the fluorescein end-label shown in A. (C) Live-cell confocal microscopy images showing the average intensities from six consecutive 1 μm slices of HeLa cells treated with peptides and polymers (*m* ∼ 60). Scale bars are 50 μm. In each study, the concentration of material is 2.5 μM with respect to fluorophore.

There are a number of non-CPP based molecular transporters capable of traversing cellular membranes with cargo in tow. These constructs are mostly comprised of a nanomaterial scaffold, such as a dendrimer, whose surface is decorated with several copies of guanidinium,^9^ the chemical moiety present on Arg side chains that endows CPPs with their cell penetrating properties.^10,11^ Near to our goal of cell penetration by peptide polymers is a strategy developed by Kiessling and co-workers in which guanidinium units are appended *via* a graft-to approach to a preformed polymer prepared by ROMP.^12,13^ This system and close derivatives designed by Tew^14^ remain the only examples of membrane penetrating polynorbornyl polymers, other than our own report^6^ describing polymerized CPPs. The strategy reported herein is inspired by these designs, but seeks a simpler, generalizable approach specific to peptide uptake. We hypothesized that incorporation of a single Arg residue into the amino acid sequence of a non-CPP, and subsequent polymerization of that peptide into a high density brush polymer, would enable cellular uptake of these materials (Fig. 1). If successful, this strategy would provide a new route to the development of peptide-based therapeutics that solves two major issues; (1) degradation by proteases and (2) inefficient cellular uptake; each of which have severely limited, if not negated, the success of many promising peptide-based drug candidates. Moreover, our strategy offers key advantages over traditional methods for conferring cellular uptake because the brush polymers produced have a much higher density (weight percentage) of the therapeutic agent and require few synthetic or purification steps.

## Results and discussion

To test our strategy, we synthesized a peptide sequence, GSGSG, that does not penetrate cells^6^ and appended one or two Arg residues to the *N* or *C* terminus, reasoning that these locations would yield the highest likelihood of maintaining the inherent bioactivity of an otherwise intact peptide sequence (Fig. 1, S1–S4 and Tables S1 and S2†). These peptides were prepared as fluorescein-labeled^15^ peptide controls and also as fluorescein-terminated brush polymers *via* graft-through ROMP^16^ (Fig. 1A). To ensure solubility, polymers were prepared as block copolymers with a second block containing an OEG (oligoethylene glycol) unit (degree of polymerization (DP) approx. 20), which does not penetrate cells alone.^6^ Note that as with any block copolymer, these materials have the potential to assemble into larger aggregates if the polymer is sufficiently amphiphilic, however, we see no evidence for the formation of such structures from these materials (for polymer synthesis and characterization data, see ESI and Fig. S5–S8 and Table S3†). Following synthesis, we then quantified the relative extent of uptake of each material in HeLa cells by flow cytometry, (Fig. 1B and S9–S11†) where concentration in these studies is with respect to fluorophore (2.5 μM) to enable direct comparison of each material's ability to transport itself and its cargo (fluorescein). In all cases, the monomeric peptide controls showed fluorescence signals that were indistinguishable from that of the vehicle control. However, peptides containing at least one Arg that were polymerized with a DP (or “*m*” in Fig. 1) of approximately 60, were able to penetrate cells as efficiently as a canonical CPP (Tat). Images from live-cell confocal microscopy supported this data, in which fluorescence signatures are observed across consecutive 1 μm *Z*-slices for only polymers containing cationic residues (Fig. 1C and S12–S17†), suggesting that these materials are internalized and not simply bound to the surface of the cell membrane.

**Fig. 2:**
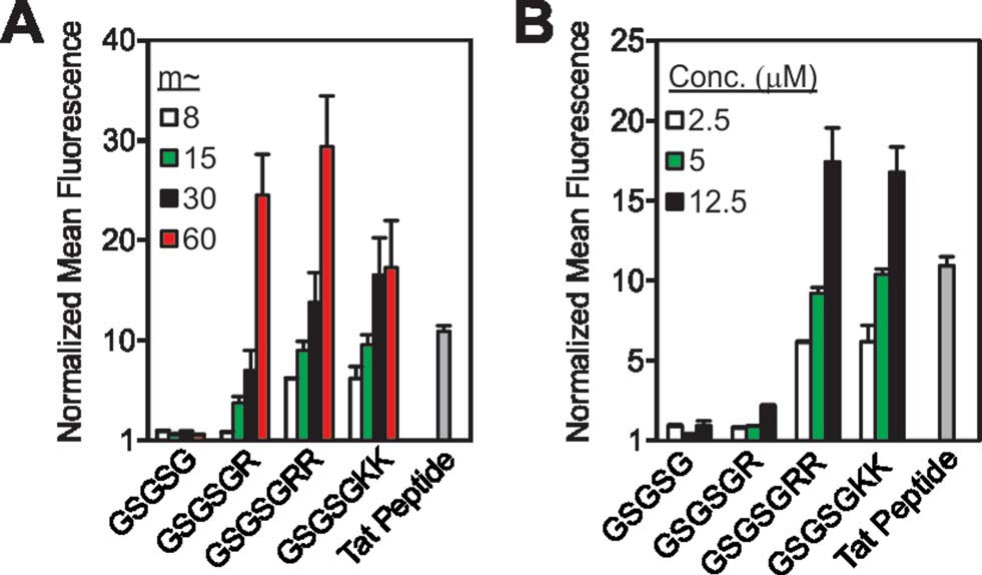
Strategies for increasing cellular uptake of GSGSG analogues. Flow cytometry data exploring the impact of (A) degree of polymerization, where each polymer is at a concentration of 2.5 μM and (B) the concentration of *m* ∼ 8 polymers. All data are normalized to DPBS at a value of 1 and concentration is with respect to fluorophore content. Data for additional polymers are shown in Fig. S10–S11.†

In general, these data reveal that peptides with Arg residues appended to the *C*-terminus (GSGSGR or GSGSGRR) exhibited better intracellular penetration than the internally buried *N*-terminal derivatives (RGSGSG or RRGSGSG) (Fig. 1B and S9–S11†). Peptides containing two Arg residues also gave more robust fluorescence signals when polymerized than those containing only one in the same position. In all cases, the Arg-containing peptide polymers gave slightly lower values than that of a polymer prepared by polymerizing a single Arg reside (R control polymer – Fig. 1B), which we consider to be the maximum theoretical signal that can result from a polymer containing one Arg per polymer side chain. In addition, peptides containing one or two lysine residues were taken up by cells when prepared as polymers but not as peptides alone, indicating that the presence of primary amino or guanidinium units was sufficient for uptake of the polymers (Fig. 1B). Moreover, the extent of uptake of each polymer was shown to be dependent upon both the degree of polymerization (Fig. 2A and S10†) and the concentration of material (Fig. 2B and S11†), suggesting that uptake of these peptides can be improved by increasing either factor.

**Fig. 3:**
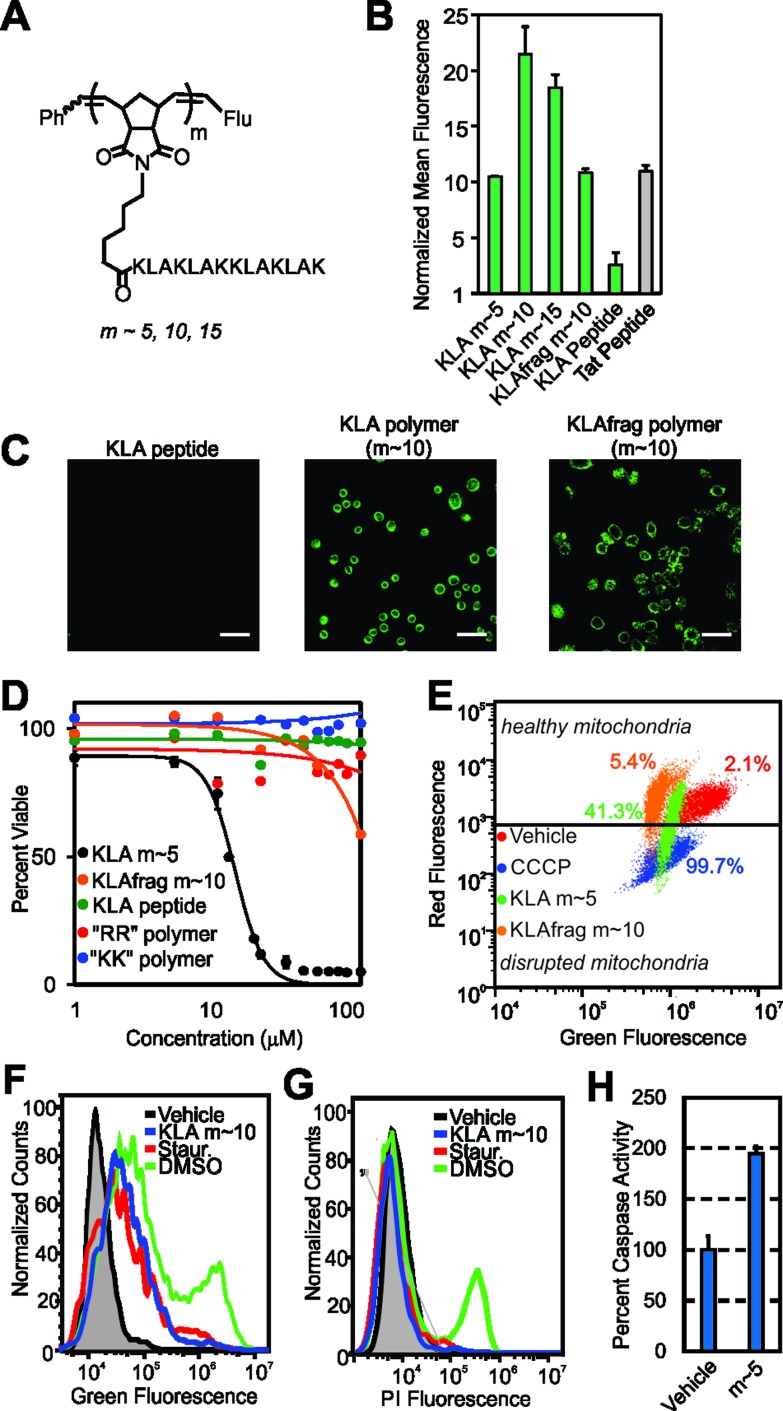
Cellular internalization and bioactivity of KLA peptide homopolymers. (A) Chemical structure of the homopolymers. “Flu” is the fluorescein end-label shown in Fig. 1A. (B) Flow cytometry data showing fluorescent signatures of HeLa cells treated with the KLA polymers and peptide. Data is normalized to DPBS at a value of 1. (C) Live-cell confocal microscopy images showing average intensities from six consecutive 1 μm slices of HeLa cells treated with the KLA peptide or polymer (*m* ∼ 10). Scale bars are 50 μm. (D) Viability of cells treated with KLA polymers (*m* ∼ 5), the KLA_fragment_ polymer (*m* ∼ 10), GSGSGRR polymer (*m* ∼ 60), GSGSGKK polymer (*m* ∼ 60) and the KLA peptide. LD_50_ values for the KLA polymers, obtained by fitting data to the Hill equation, are 12.5, 25, and 30 μM for the *m* ∼ 5, 10 and 15 polymers, respectively. Note that the dose–response curves for the *m* ∼ 10 and 15 KLA polymers are provided in Fig. S18.† (E) Mitochondrial membrane potential disruption assays. The percentages given describe the percent of signal resulting from each material in the disrupted mitochondria region. (F) Annexin V cell staining assay to identify apoptotic cells. A rightward population shift is indicative of an increase in apoptotic cells. Staurosporine (Staur.) is a known positive control for apoptosis and behaves identically to the KLA polymer in this assay (∼5-fold increase). (G) Propidium iodide cell staining assay for the identification of necrotic cells. DMSO-treated cells show a ∼40-fold increase in necrotic cells, as indicated by an increase in fluorescence of a cell population, whereas KLA polymer and staurosporine-treated cells show no shift.

Many bioactive peptides already contain one or more cationic amino acids in their sequence. Therefore, we aimed to demonstrate whether one such peptide could penetrate cells upon polymerization without the appendage of additional Arg or Lys residues. Moreover, as a crucial proof-of-concept, we aimed to determine if the peptide maintains its intended biological function when incorporated into a polymer in this manner (Fig. 3). For this purpose, we chose to use a known therapeutic peptide, KLA^17^ (sequence: KLAKLAKKLAKLAK), that does not penetrate cells at sub-millimolar concentrations despite having multiple Lys residues in its parent sequence.^18^ In previous work, KLA was shown to function by lysing cellular mitochondria, resulting in apoptosis of the cell.^17^ However, because KLA does not inherently penetrate cells, to function it must be conjugated to a CPP,^19^ prepared as a multimer,^18^ or appended to a molecular transporter.^20^

To ascertain whether KLA could penetrate cells as a polymer brush, we polymerized the peptide to varying DPs (DP or “*m*” in Fig. 3A is approx. 5, 10 and 15). At each DP, the polymers gave strong fluorescence signals by flow cytometry, similar to the Tat peptide control (Fig. 3B), whereas the KLA peptide yielded fluorescence signals indistinguishable from that of the vehicle control. Live-cell confocal microscopy verified internalization of the homopolymers at each *Z*-slice depth (Fig. 3C and S16 and S17†).

Next, to fully demonstrate the utility of our strategy, we sought to verify that the reported biological function of the KLA peptide, namely cytotoxicity by way of mitochondrial disruption, was not affected by polymerization. Validating this notion, KLA polymers demonstrated dose-dependent cytotoxicity in HeLa cells (where concentration for all cytotoxicity studies is with respect to peptide) with LD_50_ values in the range of what is seen for KLA–CPP conjugates^19,21^ (Fig. 3D and S17† for *m* ∼ 10, 15 values). Furthermore, in agreement with previous reports,^18^ no cytotoxicity was detected for the unmodified KLA peptide, presumably due to its inability to penetrate cells (Fig. 3B and C).

To confirm that the cell toxicity exhibited by the polymers was not caused by the polymer scaffold or by internalization of any cationic peptide polymer, we also performed the same assays with the GSGSG, GSGSGKK and GSGSGRR polymers (each at *m* ∼ 60). No cytotoxicity was exhibited by any of these materials at concentrations up to 1 mM. In addition, a polymer composed of a fragment of the KLA sequence (KLA_fragment_) with fewer Lys–Leu–Ala repeats (*i.e*., KLAKLAK, *m* ∼ 10), was polymerized so that the total number of amino acids was identical to that of the full length KLA polymer at *m* ∼ 5. This polymer also exhibited negligible toxicity, despite having the ability to enter cells (Fig. 3B and C). This is likely because the secondary structure of this peptide polymer, which is important for the toxicity of KLA,^22^ differed dramatically from that of the KLA peptide and its direct polymer analogue (Fig. S19†). Importantly, these data clearly indicate that the full-length amino acid sequence and secondary structure of KLA peptide is necessary for cellular toxicity of the polymers and, importantly, that a simple high density display of sequences with multiple lysines is not sufficient.

To verify that the toxicity exhibited by the KLA polymers was indeed the result of a mitochondrial dependent apoptotic process, we performed additional assays to examine the mitochondrial integrity of treated cells and to ascertain whether affected cells were early apoptotic or necrotic. In assays that probe mitochondrial integrity, cells incubated with the KLA polymers showed a decrease in healthy mitochondria and an increase in disrupted mitochondria relative to vehicle (DPBS)-treated cells (Fig. 3E). This was similar to the effects of a known small molecule positive control, carbonyl cyanide 3-chlorophenylhydrazone (CCCP). In contrast, cells treated with polymers of other cationic peptides, including the KLA_fragment_, were unaffected (Fig. 3E and S20†). Complementary cellular staining studies demonstrated that the KLA polymers caused cellular apoptosis and not necrosis as evidenced by a population-wide increase in annexin V staining, an indicator of apoptosis, as seen in Fig. 3F and no change in propidium iodide staining, Fig. 3G. These changes were similar to those exhibited by a positive control for apoptosis, staurosporine. Likewise, an increase in the expression of enzymatic markers of apoptosis, caspase 3 or 7, was seen for cells treated with KLA polymers relative to vehicle-treated cells (Fig. 3H and additional assays in Fig. S21†). Together, these data suggest that the key function of the peptide is not perturbed by polymerization.

**Fig. 4:**
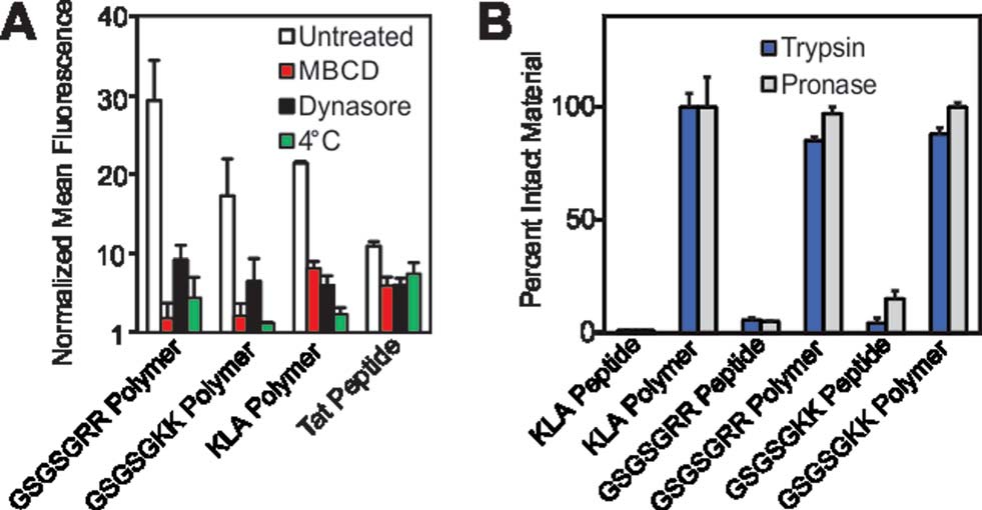
Mechanistic studies and resistance to proteases. (A) Flow cytometry data describing pharmacological inhibition of dynamin-mediated endocytosis by dynasore,^23^ membrane fluidity by methyl-β-cyclodextrin^24^ (M-βCD) or membrane trafficking by a reduction in incubation temperature. Data is normalized to DPBS at a value of 1. (B) Proteolytic susceptibility was determined by comparing RP-HPLC chromatograms of the material before and after treatment with trypsin or the protease cocktail pronase. Standard curves and individual chromatograms are provided in Fig. S22–S25.†

Having demonstrated successful cellular penetration of our materials, we next assessed the route of cellular entry by employing thermal and pharmacological inhibitors of known uptake pathways (Fig. 4A, and see ESI† for experimental details). In all cases, the uptake of the materials are similarly affected by the inhibitors tested. These data, especially the results from dynasore, an inhibitor of the key endocytosis player dynamin, suggest that polymers enter cells by endocytosis or another mechanism of membrane disruption in a manner similar to the Tat peptide.

Finally, we confirmed that these materials are resistant to proteolytic degradation. Here, GSGSGRR, GSGSGKK and KLA peptides and polymers were subjected to proteolytic digestion by trypsin and a protease cocktail (pronase). Analysis of reverse-phase HPLC (RP-HPLC) chromatograms before and after proteolytic digestion indicate that, as with our previous studies,^6^ while the peptide controls are completely degraded into fragments, the peptide polymers show little or no indication of proteolysis after incubation with multiple proteases (Fig. 4B and S22† for standard curves and S23–S25† for chromatograms). Future studies will work toward fully evaluating the utility of our polymerization strategy in living systems, including characterization of the peptide polymer's stability in serum, immunogenicity, protein binding propensities and cell selectivity.

## Experimental

### Peptide synthesis

Peptides were synthesized using standard FMOC-chemistry SPPS procedures on an AAPPTec Focus XC automated synthesizer. Peptides were prepared with protecting groups on their amino acid side chains by use of the highly acid-sensitive Sieber amide resin. If the resulting peptide was not soluble in a solvent compatible with the catalyst initiator, then the peptide was prepared protecting group free *via* use of the Rink Amide MBHA resin. Peptide monomers were synthesized by coupling the *N*-terminal residue to *N*-(hexanoic acid)-*cis*-5-norbornene-*exo*-dicarboximide.^25^ Fluorescein-labeled control peptides were assembled by addition of Boc-Lys(FMOC)-OH to the *N*-terminal residue, followed by addition of 5/6-carboxy-fluorescein after removal of the FMOC group.

### Polymerizations

All polymerizations were carried out in a glove box under N_2_ (g). A typical protocol used to generate a polymer with DP (or “*m*” in Fig. 1) = 8 involved mixing the monomer (0.0125 mmol, 8 equiv., 25 mM) with the initiator, (H_2_IMES)(pyr)_2_-(Cl)_2_Ru=CHPh,^26^ (0.00156 mmol, 1 equiv., 3.1 mM) in dry DMF (0.5 mL). To track cellular uptake, polymers were end-labeled with a copy of fluorescein by treatment with a chain transfer agent (1.5 equiv.) for 2 h as described previously,^15^ followed by termination with ethyl vinyl ether (10 equiv.) for 1 h at room temperature. Block copolymers used in the GSGSG series were prepared by first polymerizing the peptide monomer to completion prior to adding and polymerizing the OEG monomer. Following completion of the second block, the resulting polymers were end-labeled and terminated as described for the homopolymers. Polymers were subsequently characterized, and isolated as described in the ESI.†

### Cellular uptake studies

HeLa cells were cultured in Dulbecco's Modified Eagle Medium, supplemented with 10% fetal bovine serum and 1× concentrations of non-essential amino acids, sodium pyruvate, l-glutamine, and penicillin/streptomycin at 37 °C under 5% CO_2_. Cells were plated at a density of 90 000 cells per well of a 24-well plate 18 h prior to treatment. Materials dissolved in DPBS at 10× the desired concentration (where concentration is with respect to fluorophore concentration to ensure proper comparison of each molecular transporter) were added to the wells and the plates were incubated for 30 min at 37 °C. The medium was then removed and the cells were washed 2× with DPBS and then incubated 3× for five minutes each with heparin (0.5 mg mL^–1^ in DPBS) and rinsed again with DPBS. The cells were subsequently trypsinized (0.25% trypsin in DPBS) for 10 min, cold medium was added, and the cells were transferred to Eppendorf tubes. The suspended cells were centrifuged to pellets and then resuspended in a minimal amount of cold DPBS. Flow cytometry data (10 000 events on three separate cultures per condition) was then acquired.

### Cell viability assays

The cytotoxicity of materials was assessed using the CellTiter-Blue® assay. Here HeLa cells were plated at a density of 3500 cells per well of a 96-well plate 18 h prior to treatment. Non-fluorescently labeled materials dissolved in DPBS at 10× the desired concentrations were added to the wells along with a 10% DMSO positive control. Cells were incubated for 72 h at 37 °C. Note that concentration for all toxicity measurements is with respect to peptide concentration to ensure that all peptides and polymers are fairly compared with respect to their therapeutic components. The medium was removed and 80 μL of fresh medium lacking phenol red was added followed by 20 μL of the CellTiter-Blue® reagent. The cells were then incubated for 2 h prior to measuring fluorescence using 560 nm excitation and 590 nm emission.

Additional experimental details, commercial sources for all materials and supplementary figures are provided in the ESI.†

## Conclusions

In summary, we have demonstrated a new method for rendering peptides cell penetrating by incorporating them into high density polymer brushes *via* graft-through ROMP. The only requirement for successful penetration is the presence of a single Arg or Lys in the peptide sequence, preferably at the solvent-facing *C*-terminal end of the peptide. In a demonstration of the power of this strategy, we show that a known therapeutic peptide (the KLA peptide), which cannot enter cells on its own, can be rendered cell penetrating by polymerization and, importantly, maintains its sequence-specific cytotoxic function as part of a polymer. We also note that this strategy offers potential for the formulation of a therapeutic with an exceptionally high weight percentage of the active peptide (85% in our KLA homopolymer *vs.* 50% for a Tat–KLA conjugate) that is also resistant to proteolysis.^6,16^ Thus, we present a simple, effective and broadly applicable alternative to existing strategies that enable cell penetration of peptides intended for medicinal or diagnostic purposes.
