# Supplementary material for: Activating peptides for cellular uptake via polymerization into high density brushes
Source: Chem Sci. 2015 Nov 10;7(2):989–94. doi: 10.1039/c5sc03417e (PMC4763988; doi:10.1039/c5sc03417e)
Supplement: Supplementary file 1 [file SC-007-C5SC03417E-s001.pdf]

Electronic Supplementary Material (ESI) for Chemical Science.
This journal is © The Royal Society of Chemistry 2015

Supplemental Information

for

Activating Peptides for Cellular Uptake via Polymerization into High Density Brushes

Angela P. Blum,+ Jacquelin K. Kammeyer,+ and Nathan C. Gianneschi*.

Table of Contents

SI. Materials ………………………………………………………….. S3

SII. Peptide Synthesis……………………..…………………………..S3

SIII. Polymerizations……………………………………………………S7

SIV. Cell Culture…………………………………………………………..S12

SV. Flow Cytometry………………………………………………........S12

SVI. Live-Cell Confocal Microscopy…………………………………..S14

SVII. Cell Viability Assay…………………………………………….…..S17

SVIII. Circular Dichroism……………………………………...……S18

SIX. JC-1 Mitochondrial Integrity Assay…………………….……..S19

SX. Apoptosis and Necrosis Assays………………………………..S20

SXI. Apoptosis Marker Assay……………………………..…..……S21

SXII. Mechanistic Studies by Flow Cytometry………………………S21

SXIII. RP-HPLC Analysis of Proteolytic Susceptibility……..………S21

SI. Materials. All amino acids used to prepare peptides by solid phase peptide synthesis (SPPS) were obtained from AAPPTec and NovaBiochem. Unless otherwise noted, all other compounds and materials were purchased from Sigma Aldrich and used without further purification. The GSGSG monomer,[1] GSGSG peptide control,[1] Tat peptide control,[1] and OEG monomer[2] were synthesized as described previously. The polymerization initiator, **S1**, $(H_2IMES)(pyr)_2(Cl)_2Ru=CHPh$ was also prepared by a published protocol.[3] Analytical scale reverse-phase HPLC (RP-HPLC) was performed on a Jupiter Proteo90A Phenomenex column (150 × 4.60 mm) equipped with a Hitachi-Elite LaChrom L2130 pump and a UV-Vis detector (Hitachi-Elite LaChrome L-2420) monitoring at 214 nm. Peptides were purified on a preparative-scale Jupiter Proteo90A Phenomenex column (2050 × 25.0 mm) using an Armen Spot Prep II System. In all cases, peptides were purified and analyzed for purity using a gradient buffer system in which Buffer A is 0.1% TFA in water and Buffer B is 0.1% TFA in acetonitrile. Polymer dispersities (M_w/M_n) and molecular weights (M_n) were determined by size-exclusion chromatography coupled with multiangle light scattering (SEC-MALS). To this end, SEC was performed on a Phenomenex Phenogel 5 u 10, 1K-75K, 300 × 7.80 mm column in series with a Phenomenex Phenogel 5 u 10, 10K-1000K, 300 × 7.80 mm column, which ran with 0.05 M LiBr in DMF as the running buffer (flow rate of 0.75 mL/min) using a Shimadzu pump. The instrument was also equipped with a MALS detector (DAWN-HELIO, Wyatt Technology) and a refractive index (RI) detector (Wyatt Optilab T-rEX dectector). The entire SEC-MALS set-up was normalized to a 30K MW polystyrene standard. All concentrations of fluorescein-labeled materials were obtained by measuring UV absorbance of the fluorophore (at 495 nm) on a ThermoScientific Nanodrop 2000c and fitting the data obtained to the standard curves described previously.[1] The emission profiles of all materials look equivalent at these concentrations (ex: 495 nm, em: 520 nm). Fluorescent data was recorded on a Perkin Elmer EnSpire Multimode Plate Reader. A Varian Mercury Plus spectrometer was used to obtain all ^{1}H (400 MHz) NMR spectra. Chemical shifts are reported in ppm relative to the DMF-*d7* residual proton peaks. Flow cytometry was performed on an Accuri C6 flow cytometer set to default "3 blue 1 red" configuration with standard optics and slow fluidics (14 µL/min). DLS measurements were performed on a DynaPro NanoStar (Wyatt Tech). TEM images were obtained by depositing samples on carbon-formavar-coated copper grids (Ted Paella, Inc.), which were then stained with 1% w/w uranyl acetate and then imaged on a Techanai G2 Sphera operating at an accelerating voltage of 200 kV.

SII. Peptide synthesis. Peptides were synthesized using standard FMOC-chemistry SPPS procedures on an AAPPTec Focus XC automated synthesizer. Most peptide monomers were synthesized to contain amino acid side chain protecting groups by the use of the highly acid-sensitive Sieber Amide resin, which allows for cleavage of the peptide from the resin without removal of the protecting groups. Peptides that were not soluble in DMF when protected were prepared protecting group-free via the use of Rink Amide MBHA resin. For details on the amino acid sequence and protecting groups used, see Figure S1 for structures.

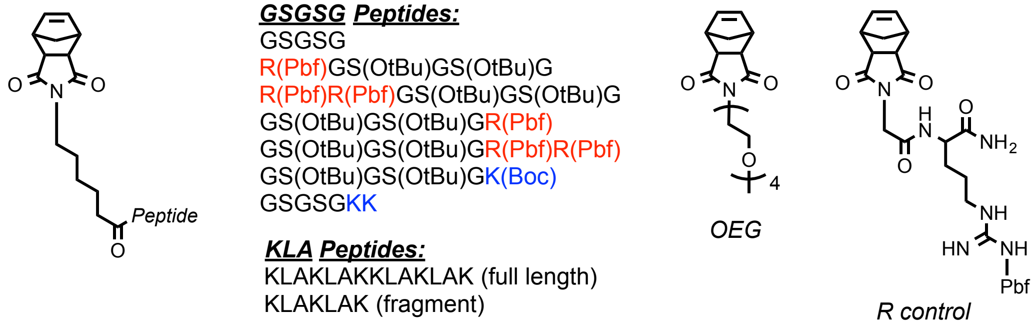

Figure S1. Chemical structures of ROMP monomers. The GSGSGKK peptide and all KLA variants were prepared without protecting groups for enhanced solublity in DMF in preparation for polymerization.

A typical SPPS procedure involved deprotection of the *N*-terminal FMOC group with 20 % methyl-piperidine in DMF (1 × 5 min, followed by 1 × 15 min), and 45 min amide couplings using 3.75 equiv. of the protected amino acid, 4 equiv. of HBTU and 8 equiv. of DIPEA. Peptide couplings that were unsuccessful by Kaiser test were drained and then subjected to fresh reagents. Monomers were prepared by amide coupling to *N*-(hexanoic acid)-*cis*-5-norbornene-*exo*-dicarboximide (described previously)[4] at the *N*-terminus of the peptide. The "R control" was prepared by conjugating Arg to *N*-(glycine)-*cis*-5-norbornene-*exo*-dicarboximide.[5] The *N*-glycine derivative was used to produce a shorter linker between the Arg residue and the norbornene unit to provide as little flexibility as possible. Data from this control is, thus, taken to reflect the maximum theoretical uptake that should be achieved by a peptide containing a single Arg. Fluorescein-labeled peptides were assembled by addition of Boc-Lys(FMOC)-OH to the *N*-terminus of the peptide, followed by removal of the FMOC protecting group and, finally, amide coupling to 5/6-carboxy fluorescein using the conditions described above. Following completion of the synthesis, peptides were cleaved from the resin. All side-chain protected peptide monomers were cleaved from

the Sieber amide resin by five consecutive rinses with 2% TFA in DCM for two minutes each. All other peptides were cleaved from Rink MBHA resin and deprotected by treatment with TFA/H_2O/TIPS in a 9.5:2.5:2.5 ratio for 2 hrs. The peptides were then precipitated in cold ether and purified by RP-HPLC. The purity of each peptide was verified by analytical RP-HPLC, where a single peak in the chromatogram of a newly purified peptide was taken as an indication of a pure material (Figures S2-3). The identity of each peptide was confirmed by ESI MS (Tables S1-2).

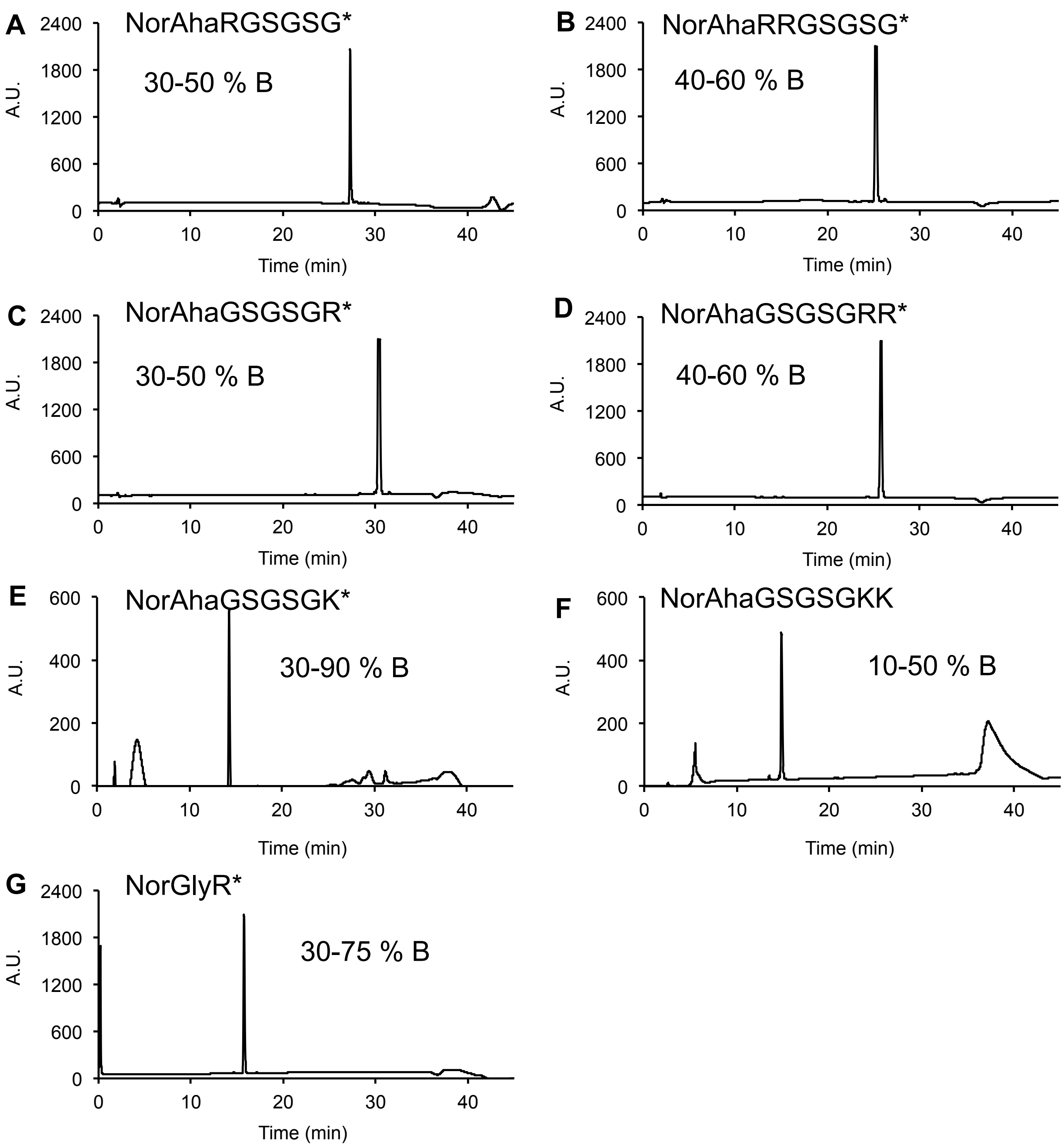

Figure S2. RP-HPLC chromatograms confirming the purity of peptide monomers. NorAha = *N*-(hexanoic acid)-*cis*-5-norbornene-*exo*-dicarboximide; NorGly = *N*-(glycine)-*cis*-5-norbornene-*exo*-dicarboximide. Note that the peptides given in A-E and G were synthesized with protecting groups on the amino acid side chains. Chromatograms are included for the following: (A) NorAhaR(Pbf)GS(OtBu)GS(OtBu)G-NH_2 at 30-50% Buffer B, (B) NorAhaR(Pbf)R(Pbf)GS(OtBu)GS(OtBu)G-NH_2 at 40-60% Buffer B, (C) NorAhaGS(OtBu)GS(OtBu)GR(Pbf)-NH_2 at 30-50% Buffer B (D) NorAhaGS(OtBu)GS(OtBu)GR(Pbf)R(Pbf)-NH_2 at 40-60% Buffer B, (E) NorAhaGS(OtBu)GS(OtBu)GK(Boc)-NH_2 at 30-90% Buffer B, (F) NorAhaGSGSGKK-NH_2 at 10-50% Buffer B, (G) NorGlyR-NH_2 at 30-75% Buffer B. The identity of each peak was confirmed by ESI MS as shown in Table S1.

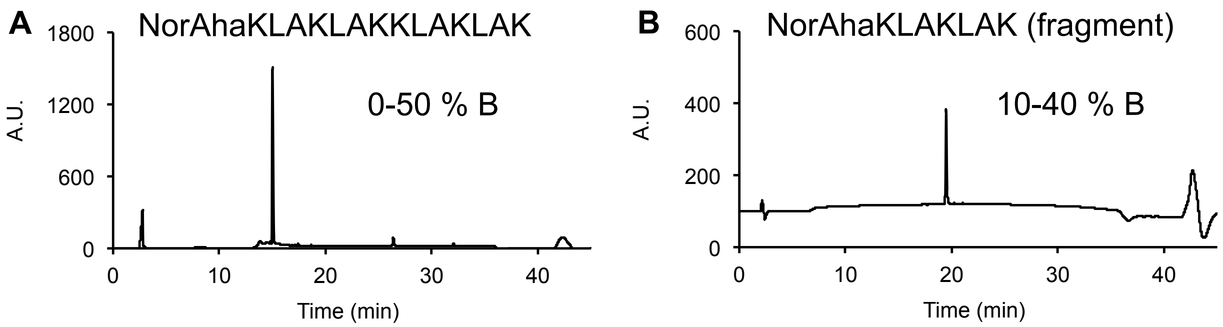

Figure S3. RP-HPLC chromatograms confirming the purity of KLA peptide monomers. NorAha = *N*-(hexanoic acid)-*cis*-5-norbornene-*exo*-dicarboximide; NorGly = *N*-(glycine)-*cis*-5-norbornene-*exo*-dicarboximide. Chromatograms are included for the following: (A) NorAhaKLAKLAKKLAKLAK-NH_2 (full length KLA) at 0-50% Buffer B, (B) NorAhaKLAKLAK-NH_2 (fragment KLA) at 10-40% Buffer B. The identity of each peak was confirmed by ESI MS as shown in Table S1.

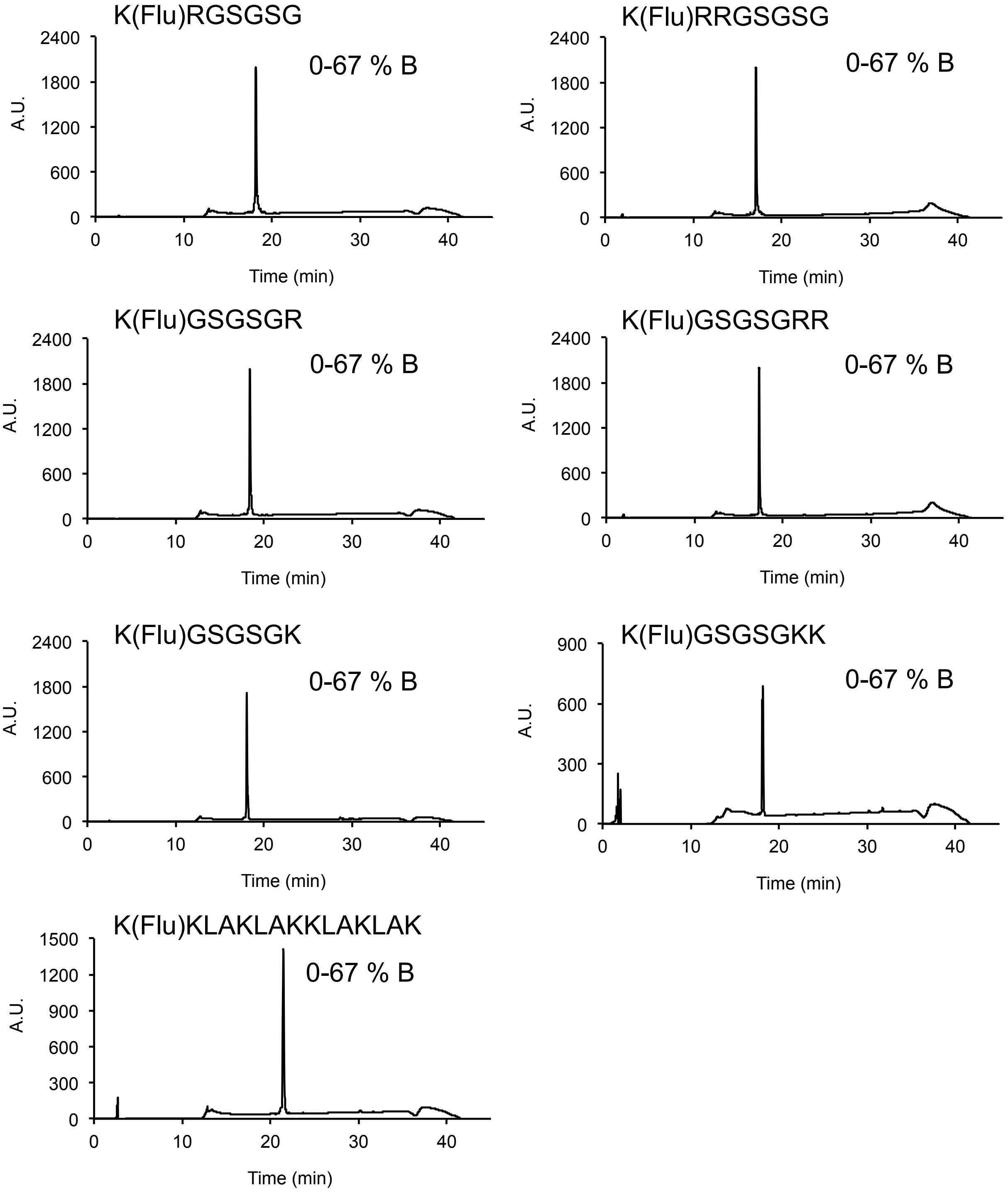

Figure S4. RP-HPLC chromatograms confirming the purity of fluorescein-labeled peptide controls. Each peptide was purified using a gradient of 0-67% Buffer B. The identity of each peak was confirmed by ESI MS as shown in Table S2. Flu represents 5/6-carboxyfluorescein, which is conjugated to the ε amino group of the N-terminal Lys on each peptide.

Peptide Monomer	Calculated Mass	Obtained Mass (ESI)
NorAhaR(Pbf)GS(OtBu)GS(OtBu)G-NH_2	1142	1143
NorAhaR(Pbf)R(Pbf)GS(OtBu)GS(OtBu)G-NH_2	1550	1552
NorAhaGS(OtBu)GS(OtBu)GR(Pbf)-NH_2	1142	1143
NorAhaGS(OtBu)GS(OtBu)GR(Pbf)R(Pbf)-NH_2	1550	1552
NorAhaGS(OtBu)GS(OtBu)GK(Boc)-NH_2	964	963
NorAhaGSGSGKK-NH_2	879	878
NorAhaKLAKLAKKLAKLAK-NH_2 (full length)	1781	1782
NorAhaKLAKLAK-NH_2 (fragment)	1029	1027
NorGlyR(Pbf)-NH_2	628	627

Table S1. Molecular masses obtained for each peptide monomer. Note that several sequences were synthesized with protecting groups on the amino acid side chains. Masses were obtained by ESI MS and confirm the identity of each monomer.

Peptide Control	Calculated Mass	Obtained Mass (ESI)
K(Flu)RGSGSG-NH_2	1005	1004
K(Flu)RRGSGSG-NH_2	1161	1160
K(Flu)GSGSGR-NH_2	1005	1004
K(Flu)GSGSGRR-NH_2	1161	1160
K(Flu)GSGSGK-NH_2	977	978
K(Flu)GSGSGKK-NH_2	1106	1105
K(Flu)KLAKLAKKLAKLAK-NH_2	2011	2010

Table S2. Molecular masses obtained for each fluorescently-labeled peptide control. All sequences are prepared with an *N*-terminal Lys that is conjugated to 5/6-carboxyfluorescein via the ε-amino group. Masses were obtained by ESI MS and confirm the identity of each monomer.

SIII. Polymerizations. All polymerizations were carried out in a glove box under N_2 (g). A typical protocol (Figures S4-5) used to generate a polymer with DP (or "m" in Figure 1 of the main text) = 8 involved mixing the monomer (0.0125 mmol, 8 equiv., 25 mM) with the catalyst initiator **S1** (0.00156 mmol, 1 equiv., 3.1 mM) in dry DMF (0.5 mL). For each peptide monomer whose polymerization has not been reported previously in the literature, we followed the time course of the polymerization in DMF-*d7* by ^{1}H NMR to confirm complete consumption of the monomer and to determine the time period required to reach completion (Figure S6). To track cellular uptake, each polymer was end-labeled with a copy of fluorescein by treatment with a chain transfer agent (1.5 equiv.) for 2 hrs as described previously,[2] followed by termination with ethyl vinyl ether (10 equiv.) for 1 hr at room temperature. Block copolymers used in the GSGSG series were prepared by first polymerizing the peptide monomer to completion prior to adding and polymerizing the OEG monomer (Figure S4). The block copolymers were prepared in this manner to obtain an accurate estimation of the DP or m of the functional peptide block (using a dn/dc of 0.179 in DMF) since the peptide and OEG blocks have different dn/dc values in DMF. The accessory, water-solubilizing OEG block (dn/dc = 0.11 in DMF) was then polymerized second and ratios of the dn/dc values of the two blocks were used to calculate values for this block (*i.e.*, the dn/dc for m = 8, 15, 30 and 60 was 0.131, 0.141, 0.151 and 0.161 based on the ratio of peptide:OEG used). Following completion of the second block, the resulting polymers were end-labeled with the fluorescein chain transfer agent and the active catalyst was then terminated with ethyl vinyl ether as described above. Polymerization reactions of peptides intended for use in cytotoxicity studies were split in half prior to addition of the fluorescein end label. One half was then labeled with the fluorescein chain transfer agent and terminated as described (for use in cell-uptake studies) and the second half was terminated directly with ethyl vinyl ether (for use in toxicity studies). Fluorescein-labeled polymers were treated with NH_4OH (aq) for 30 min to remove the pivolate protecting group, as described previously.[2] The resulting polymers were then directly characterized by SEC-MALS to obtain the polymer molecular weight (M_n), dispersity and degree of polymerization (DP).

All polymers were then precipitated with cold ether and collected by centrifugation. For the side-chain protected peptide monomers, the resulting powder was dissolved in 2 mL of a mixture of TFA/H_2O/TIPS (95:2.5:2.5) and stirred for 4 hours at room temperature. The product was precipitated with cold ether, collected by centrifugation

and dried. In preparation for *in vitro cell* studies, all polymers were washed (3×) with cold ether (to remove the Ru catalyst) and then dissolved in DPBS and dialyzed in dialysis cups with a molecular weight cut-off value of 3500 (Thermo Scientific, cat. # 69552) in an effort to remove any residual monomer or catalyst.

We also sought to determine whether our block copolymers (*i.e.,* GSGSGRR$_{60}$-*b*-OEG$_{20}$ and derivatives) formed larger aggregate structures in aqueous solution. To this end, we characterized three polymers GSGSGRR m ~ 60, GSGSGKK m ~ 60 and GSGSG m ~ 60 dissolved in DPBS by standard dynamic light scattering (DLS) and transition electron microscopy (TEM) analyses, which we perform routinely in other work to characterize nano- and micron-scale polymer assemblies. For each material (at 10 x the concentration used in uptake studies), diameters of <6 nm were observed by DLS, which correlate to unimers according to the rough aggregation numbers calculated by the instrument. Likewise, TEM images were indistinguishable from that of the buffer (DPBS) alone. While it is difficult to ever rule out aggregation as a possibility, we see no clear evidence for the formation of such structures. We also note that, in general, aggregation of any peptide polymer, including homopolymers, is possible and will likely depend heavily on the amino acid sequence used and its arrangement on the polymer backbone.

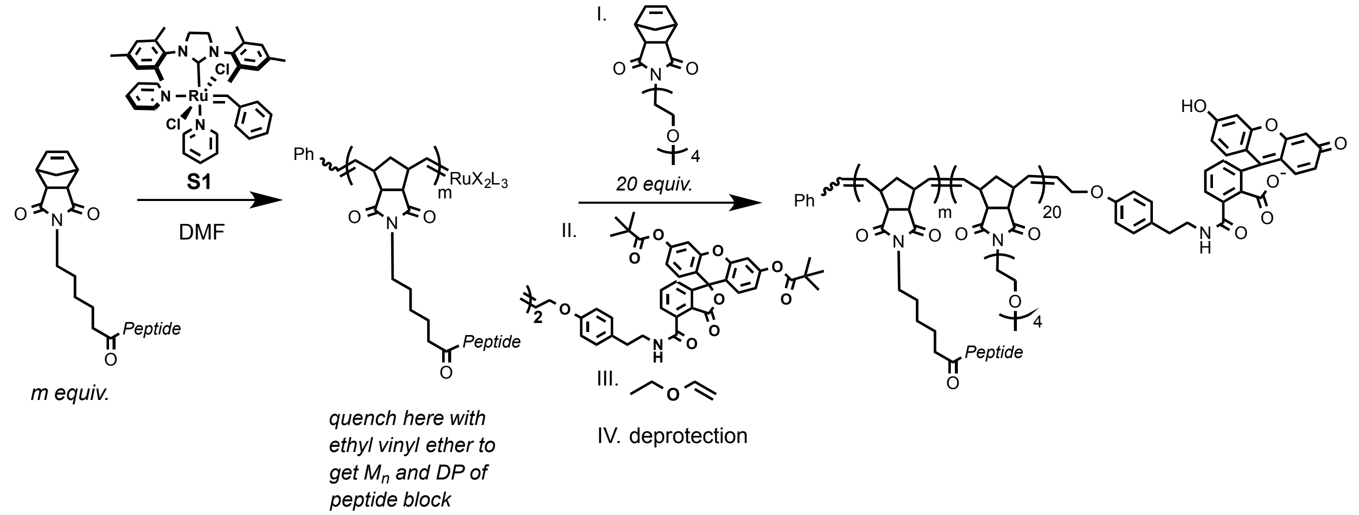

Figure S5. Polymerization scheme for the preparation of GSGSG peptide polymers and analogues. Peptide polymers for this series are prepared via ROMP as block copolymers with an accessory OEG block to ensure aqueous solubility of all GSGSG analogues. Each peptide block is polymerized to m ~ 8, 15, 30, and 60. The OEG block is kept constant at a DP of ~ 20. The living polymer can be terminated with ethyl vinyl ether after completion of the peptide block to gain accurate molecular weights (M_n), dispersity (M_w/M_n) and degree of polymerization (DP). To monitor uptake of the materials, each polymer is end-labeled with fluorescein. Polymers used in all toxicity studies, were prepared without a fluorophore by terminating the polymerization with ethyl vinyl ether after addition of the second (OEG) block.

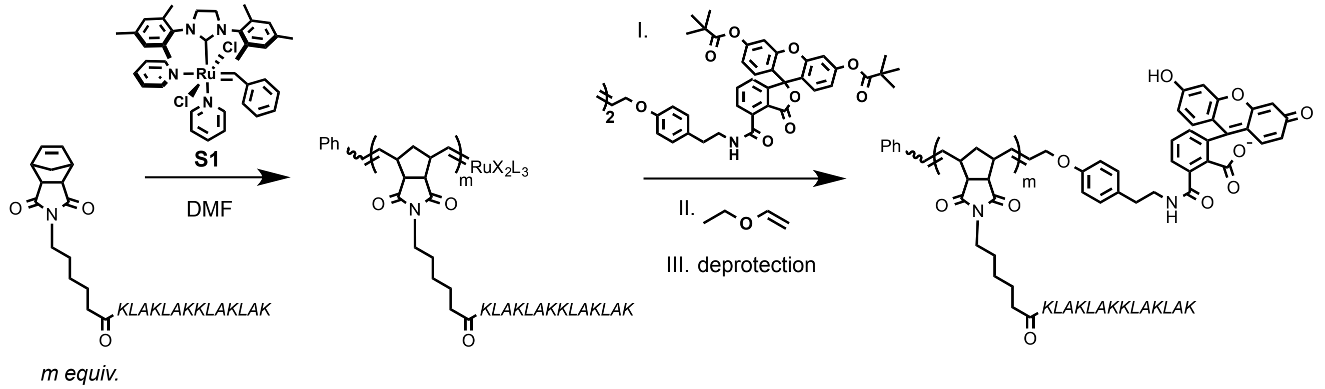

Figure S6. Polymerization scheme for the preparation of KLA homopolymers. KLA peptide polymers were prepared as homopolymers (m ~ 5, 10, 15) via ROMP and end-labeled with fluorescein as with the block copolymers described in Figure S5. Identical conditions were used to prepare the KLAKLAK (fragment) control polymer. Polymers used in all toxicity studies, were prepared without a fluorophore by terminating the polymerization with ethyl vinyl ether after the peptide finished polymerizing.

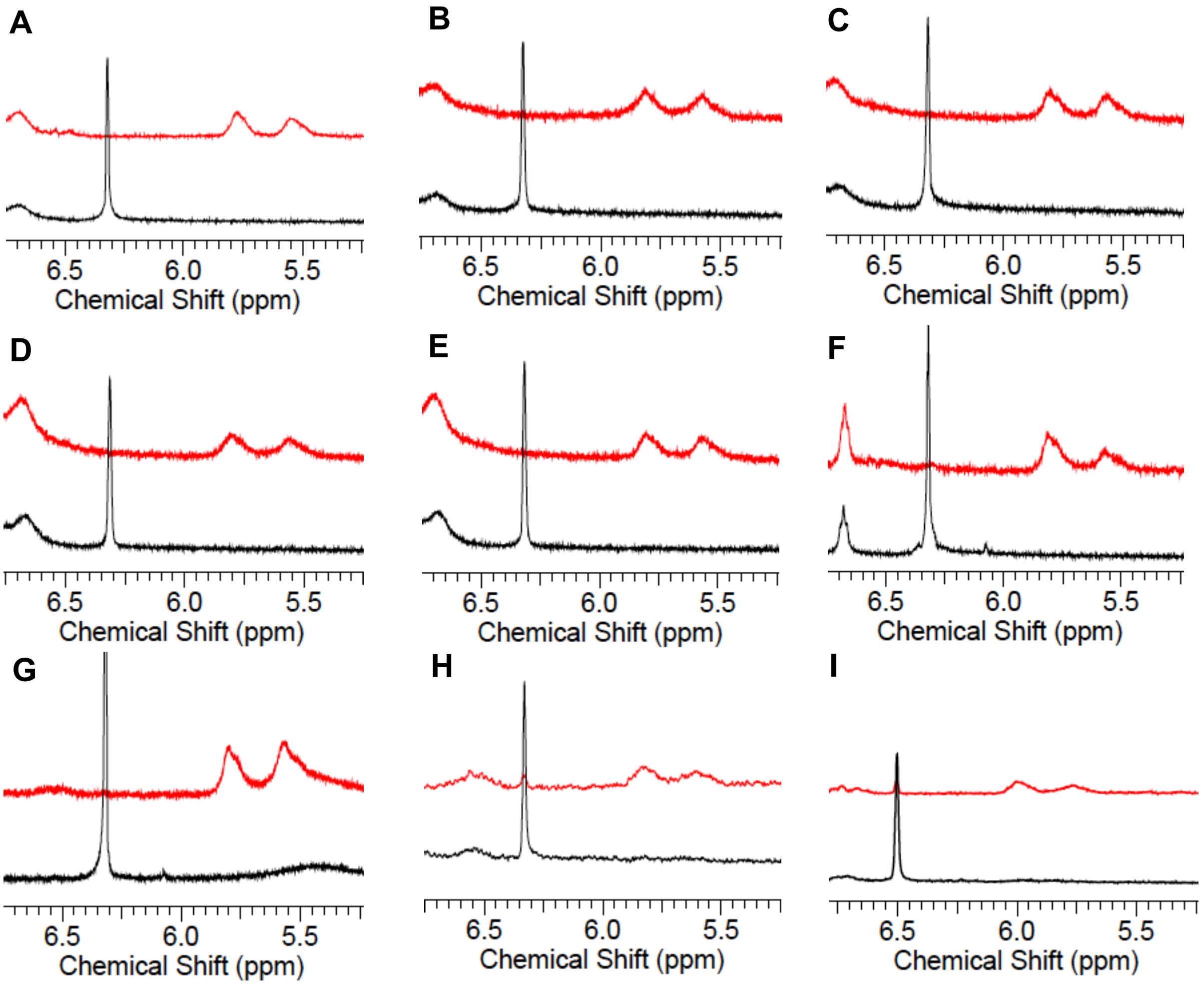

Figure S7. ^{1}H NMR time course spectra for the polymerization of all monomers that have not been reported previously in the literature: (A) R control (B) GSGSGR (C) GSGSGR (D) RRGSGSG (E) GSGSGRR (F) GSGSGK (G) GSGSGKK (H) KLA (full length), (I) KLA (fragment). Polymers are polymerized to a DP (m) of ~ 10. Black spectra were taken at t = 0 and red spectra were taken at t = 3 hr. Note the disappearance of the resonance at δ = 6.32 ppm corresponding to the olefin protons of the monomer and the coincident appearance of resonances at δ = 5.5−6 ppm, which correspond to the *cis* and *trans* olefin protons of the polymer backbone.

		First Block (Peptide Block)			Second Block (OEG Block)		
Polymer Name in Text[a]	Polymer IUPAC[b]	M_n[c]	M_w/M_n[d]	DP[e] ("m")	M_n[c]	M_w/M_n[d]	DP[e]
GSGSG, m ~ 8	$GSGSG_{11}$-*b*-OEG_{18}	6,560	1.012	11 (8)	12,800	1.12	18 (20)
GSGSG, m ~ 15	$GSGSG_{16}$-*b*-OEG_{21}	9,800	1.108	16 (15)	17,000	1.054	21 (20)
GSGSG, m ~ 30	$GSGSG_{28}$-*b*-OEG_{16}	17,700	1.125	28 (30)	23,500	1.075	16 (20)
GSGSG, m ~ 60	$GSGSG_{56}$-*b*-OEG_{28}	35,000	1.274	56 (60)	45,100	1.052	28 (20)
R control, m ~ 8	R_{12}-*b*-OEG_{15}	7700	1.17	12 (8)	13,000	1.101	15 (20)
R control, m ~ 15	R_{18}-*b*-OEG_{27}	11,700	1.009	19 (15)	21,500	1.061	27 (20)
R control, m ~ 30	R_{26}-*b*-OEG_{20}	16,800	1.027	27 (30)	23,700	1.048	20 (20)
R control, m ~ 60	R_{52}-*b*-OEG_{20}	33,900	1.036	54 (60)	40,800	1.046	20 (20)
RGSGSG, m ~ 8	$RGSGSG_{10}$-*b*-OEG_{21}	11,600	1.005	10 (8)	19,300	1.015	21 (20)
RGSGSG, m ~ 15	$RGSGSG_{15}$-*b*-OEG_{16}	16,500	1.09	15 (15)	22,230	1.06	16 (20)
RGSGSG, m ~ 30	$RGSGSG_{29}$-*b*-OEG_{27}	33,100	1.007	29 (30)	42,800	1.014	27 (20)
RGSGSG, m ~ 60	$RGSGSG_{68}$-*b*-OEG_{26}	78,000	1.143	68 (60)	90,300	1.142	26 (20)
GSGSGR, m ~ 8	$GSGSGR_{11}$-*b*-OEG_{22}	12,130	1.08	11 (8)	20,000	1.034	22 (20)
GSGSGR, m ~ 15	$GSGSGR_{18}$-*b*-OEG_{18}	20,000	1.034	18 (15)	26,300	1.085	18 (20)
GSGSGR, m ~ 30	$GSGSGR_{26}$-*b*-OEG_{14}	30,100	1.053	26 (30)	35,000	1.025	14 (20)
GSGSGR, m ~ 60	$GSGSGR_{71}$-*b*-OEG_{26}	81,100	1.151	71 (60)	90,300	1.142	26 (20)
RRGSGSG, m ~ 8	$RRGSGSG_{9}$-*b*-OEG_{16}	14,500	1.018	9 (8)	20,000	1.018	16 (20)
RRGSGSG, m ~ 15	$RRGSGSG_{15}$-*b*-OEG_{28}	23,800	1.018	15 (15)	33,700	1.019	28 (20)
RRGSGSG, m ~ 30	$RRGSGSG_{31}$-*b*-OEG_{14}	48,500	1.006	31 (30)	53,400	1.007	14(20)
RRGSGSG, m ~ 60	$RRGSGSG_{52}$-*b*-OEG_{29}	80,700	1.001	52 (60)	90,800	1.003	29 (20)
GSGSGRR, m ~ 8	$GSGSGRR_{8}$-*b*-OEG_{19}	12,500	1.052	8 (8)	19,200	1.014	19 (20)
GSGSGRR, m ~ 15	$GSGSGRR_{18}$-*b*-OEG_{23}	28,500	1.056	18 (15)	36700	1.078	23(20)
GSGSGRR, m ~ 30	$GSGSGRR_{30}$-*b*-OEG_{19}	47,000	1.27	30 (30)	48,200	1.041	19 (20)
GSGSGRR, m ~ 60	$GSGSGRR_{57}$-*b*-OEG_{13}	89,000	1.103	57 (60)	93,600	1.053	13 (20)
GSGSGK, m ~ 8	$GSGSGK_{10}$-*b*-OEG_{23}	9,650	1.047	10 (8)	17,900	1.044	23 (20)
GSGSGK, m ~ 15	$GSGSGK_{13}$-*b*-OEG_{16}	12,100	1.022	13 (15)	17,800	1.16	16 (20)
GSGSGK, m ~ 30	$GSGSGK_{26}$-*b*-OEG_{17}	24,800	1.056	26 (30)	30,700	1.042	17 (20)
GSGSGK, m ~ 60	$GSGSGK_{53}$-*b*-OEG_{20}	50,800	1.114	53 (60)	57,900	1.165	20 (20)
GSGSGKK, m ~ 8	$GSGSGKK_{8}$-*b*-OEG_{22}	7,170	n.d.	8 (8)	14,900	n.d.	22 (20)
GSGSGKK, m ~ 15	$GSGSGKK_{16}$-*b*-OEG_{17}	13,700	n.d.	16 (15)	19,800	n.d.	17 (20)
GSGSGKK, m ~ 30	$GSGSGKK_{33}$-*b*-OEG_{19}	29,000	n.d.	33 (30)	35,600	n.d.	19 (20)
GSGSGKK, m ~ 60	$GSGSGKK_{61}$-*b*-OEG_{30}	54,000	n.d.	61 (60)	64,500	n.d.	30 (20)
KLA full length, m ~ 5	KLA_{6}	10,300	n.d.	6 (5)	-	-	-
KLA full length, m ~ 10	KLA_{11}	20,200	n.d.	11(10)	-	-	-
KLA full length, m ~ 15	KLA_{14}	25,000	n.d.	14 (15)	-	-	-
KLA fragment, m ~ 10	$KLA(fragment)_{11}$	11,200	n.d.	11(10)			

Table S3. GSGSG-derived block copolymers and KLA homopolymers. [a]The name of the polymer as referred to in the text. Note that the approximate degree of polymerization (DP) is given to best compare similarly sized polymers. Also note that the OEG coblock is omitted from the name. [b]The IUPAC name of the polymer with the actual DPs of blocks in the block copolymer. All polymers are end-labeled with a copy of fluorescein. [c]Number average molecular weight, except for GSGSGKK and KLA polymers, which are weight average molecular weight (M_w) [d]Dispersity of each block. [e]Experimentally determined DPs with the theoretical DPs, based on the initial monomer-to-initiator ratio, given in parentheses. These values were obtained by SEC-MALS except for those describing the GSGSGKK and KLA polymers, which did not elute on the SEC column. All values for these polymers were determined by batch-mode static light scattering without the SEC component. As such, no information on the dispersity of these polymers was obtained. The SEC-MALS chromatogram for all m ~ 60 GSGSG derivatives that do elute on the SEC column are provided in Figure S7. All peptide values are calculated with a dn/dc of 0.179.

The OEG block has a dn/dc of 0.11 and so a ratio (based on the initial monomer-to-OEG ratio) was used to calculate values for this block: the dn/dc from m ~ 8, 15, 30 and 60 were 0.131, 0.141, 0.151 and 0.161, respectively.

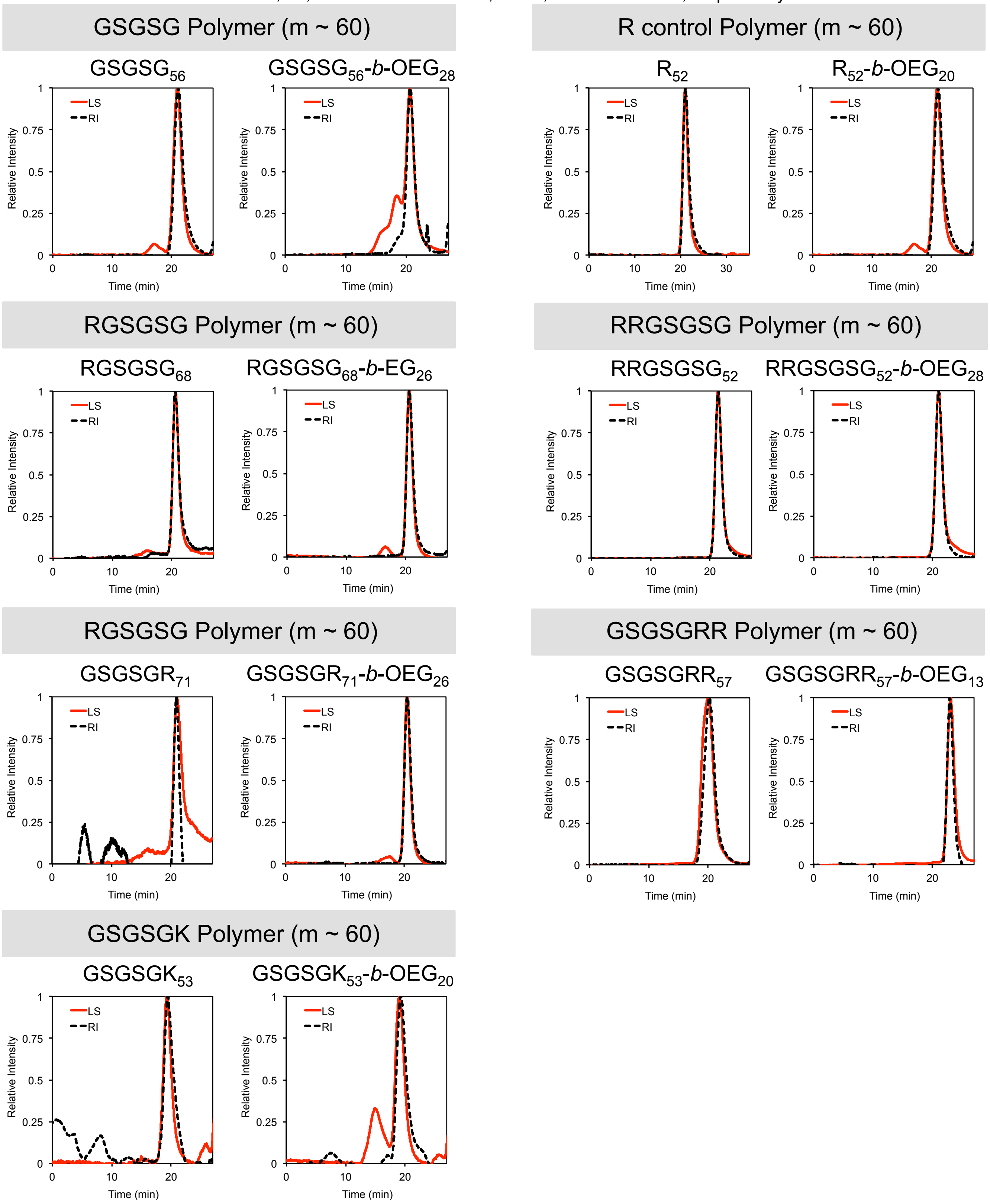

Figure S8. SEC-MALS chromatograms for GSGSG polymers and analogues at a peptide m of ~ 60 in DMF with 0.05 M LiBr. In some traces, a multimodal distribution is seen by LS, but only a monomodal distribution is observed at t > 15 m in the RI component. In these scenarios, only the peak width with an associated RI component is used to calculate values. Note that the GSGSGK monomer with side chain protecting groups formed a gel upon polymerization, which made it difficult to run these samples on the SEC column. A saturated solution of LiBr was added to break up the gel to achieve the traces shown. The deprotected polymer did not aggregate in DPBS and showed no signal by dynamic light scattering, suggesting that no larger aggregates were present.

SIV. Cell culture. HeLa cells were purchased from ATCC (CCL-2). Cells were cultured at 37 ° C under 5% CO_2 in Dulbecco's Modified Eagle Medium containing phenol red (DMEM; Gibco Life Tech., cat # 11960-044) that was supplemented with 10% fetal bovine serum (Omega Scientific, cat # FB02) and with 1× concentrations of non-essential amino acids (Gibco Life Tech., cat # 11140-050) sodium pyruvate (Gibco Life Tech., cat. # 11360-070), L-Glutamine (Gibco Life Tech., cat. # 35050-061), and the antibiotics penicillin/streptomycin (Corning Cellgro, cat. # 30-002-C1). Cells were grown in T75 culture flasks and subcultured at ~75−80% confluency (every ~ 3−4 days).

SV. Flow cytometry. HeLa cells were plated at a density of 90,000 cells per well of a 24-well plate 18 hrs prior to treatment with the material of interest. Materials dissolved in Dulbecco's Phosphate Buffered Saline (DPBS without Ca^{2+} or Mg^{2+}; Corning Cellgro, cat. # 21-031-CM) at 10× the desired concentration (where concentration is with respect to fluorophore concentration to ensure propar comparison of each molecular transporter) were added to the wells and the plates were incubated for 30 min at 37 °C. The medium was then removed and the cells were washed 2× with DPBS and then incubated 3× for five minutes each with heparin (0.5 mg/mL in DPBS; Affymetrix, cat. # 16920) to remove any membrane-bound, non-internalized material as described by Dowdy,[6] and finally rinsed again with DPBS. The cells were then trypsinized (0.25 % trypsin in DPBS; Gibco Life Tech., cat. # 15090-046) for 10 min, cold medium was added, and the cells were transferred to Eppendorf tubes. The suspended cells were centrifuged to pellets and then resuspended in a minimal amount of cold DPBS. Flow cytometry data (10,000 events on three separate cultures per condition) was then acquired.

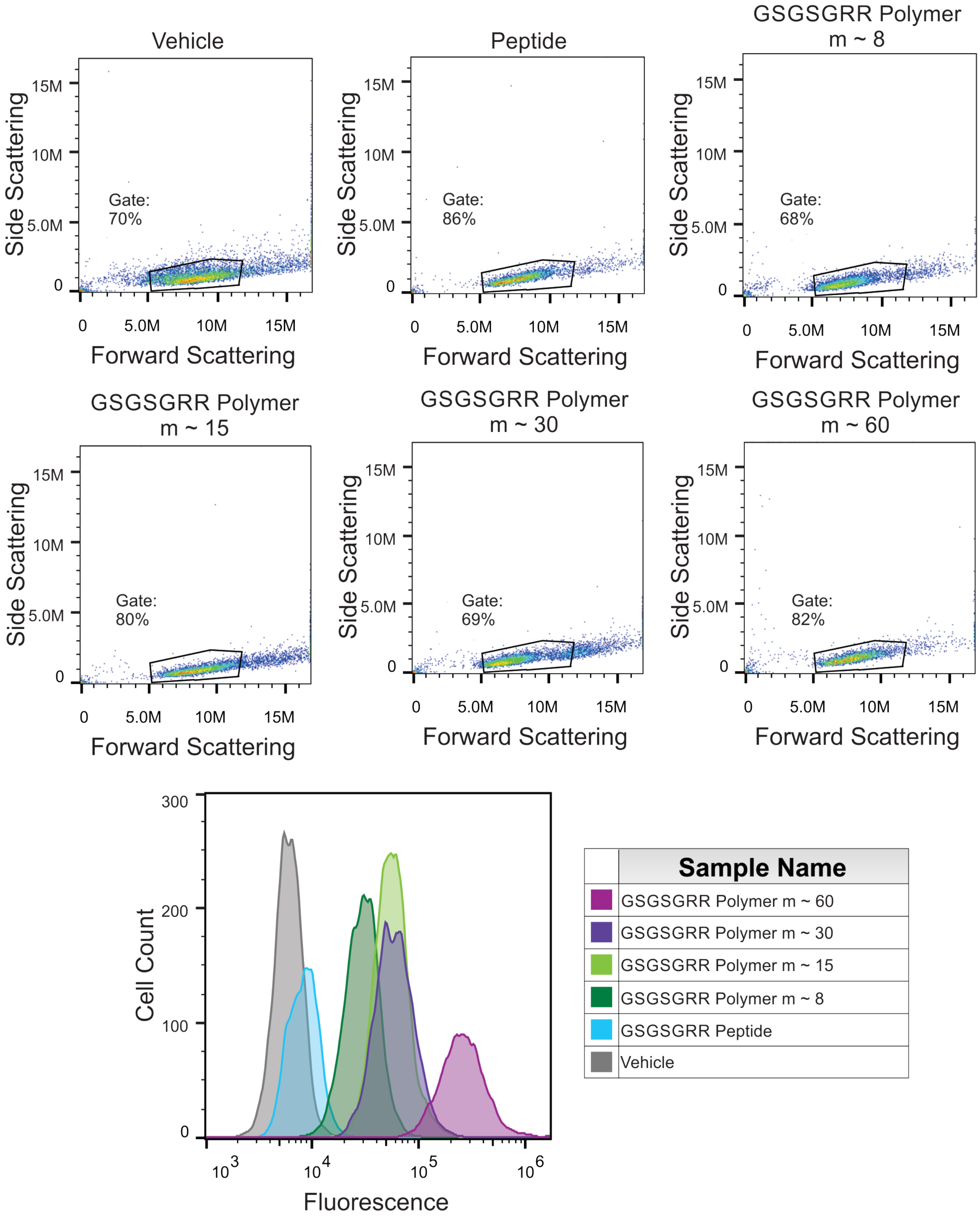

Figure S9. Example flow cytometry data for GSGSGRR. Healthy populations (10,000 events each) were gated identically and referenced to vehicle (DPBS). Histograms describing fluorescence observed for each material are shown. Each measurement was repeated 3× on three separate cultures. The concentration for each material is 2.5 μM, where concentration is with respect to the fluorophore.

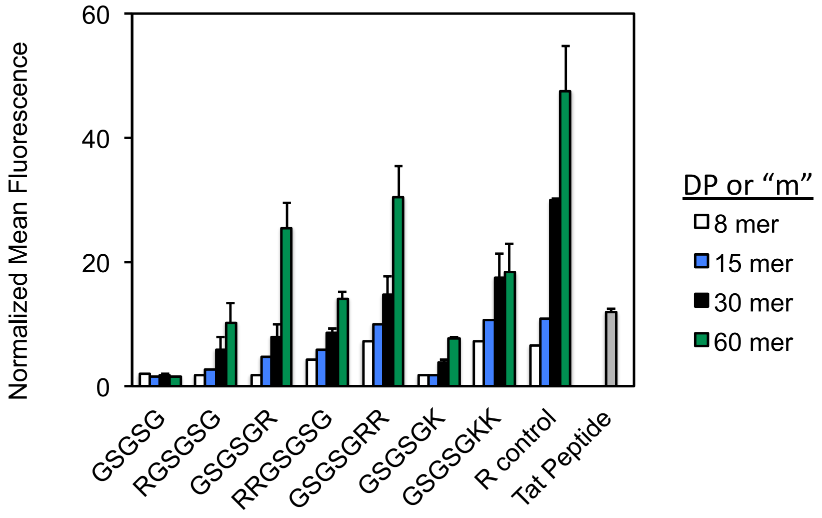

Figures S10. Influence of the degree of polymerization or "m" on cellular uptake as quantified by flow cytometry. All data are referenced to vehicle (DPBS), which gives a value of 1. The concentration for each material is 2.5 μM, where concentration is with respect to the fluorophore.

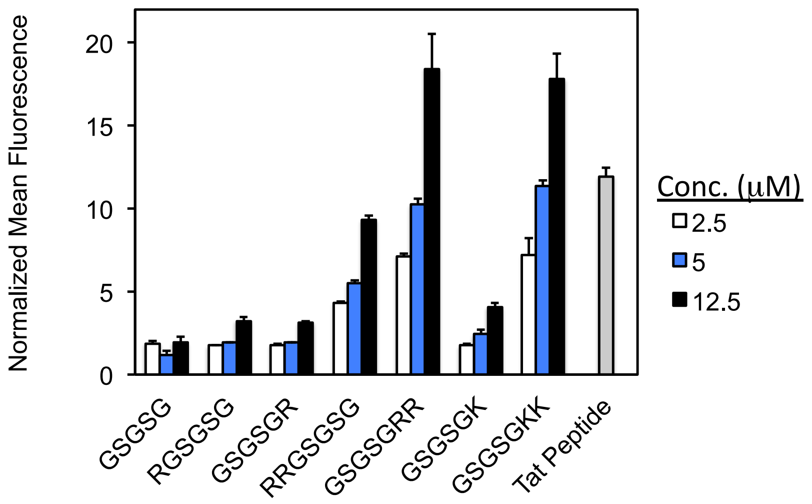

Figures S11. Concentration dependence of cellular uptake as quantified by flow cytometry. All data are referenced to vehicle (DPBS), which gives a value of 1. Concentration of all materials is with respect to the fluorophore.

SVI. Live-cell confocal microscopy. HeLa cells were plated on glass-bottom 24-well plates at a cell density of 90,000 cells per well 18 hrs prior to treatment with the compound of interest. The medium was removed and then replaced with medium lacking phenol red (Gibco Life Tech., cat# 31053-028) to minimize background fluorescence. Materials dissolved in DPBS (at 10× the desired concentration, 2.5 μM, where concentration is with respect to fluorophore) were added to the wells and the plates were incubated for 30 min at 37 °C. The medium was then removed and the cells were rinsed 2× with DPBS, and then fresh medium (phenol red- free) was added to each well. Live cells were imaged on an Olympus FV1000 confocal microscope with a chamber at 37 ° C under 5% CO_2 via a Z-stack analysis (set to 1 μm slices) using identical instrument settings for each image and a 40× or 100x objective.

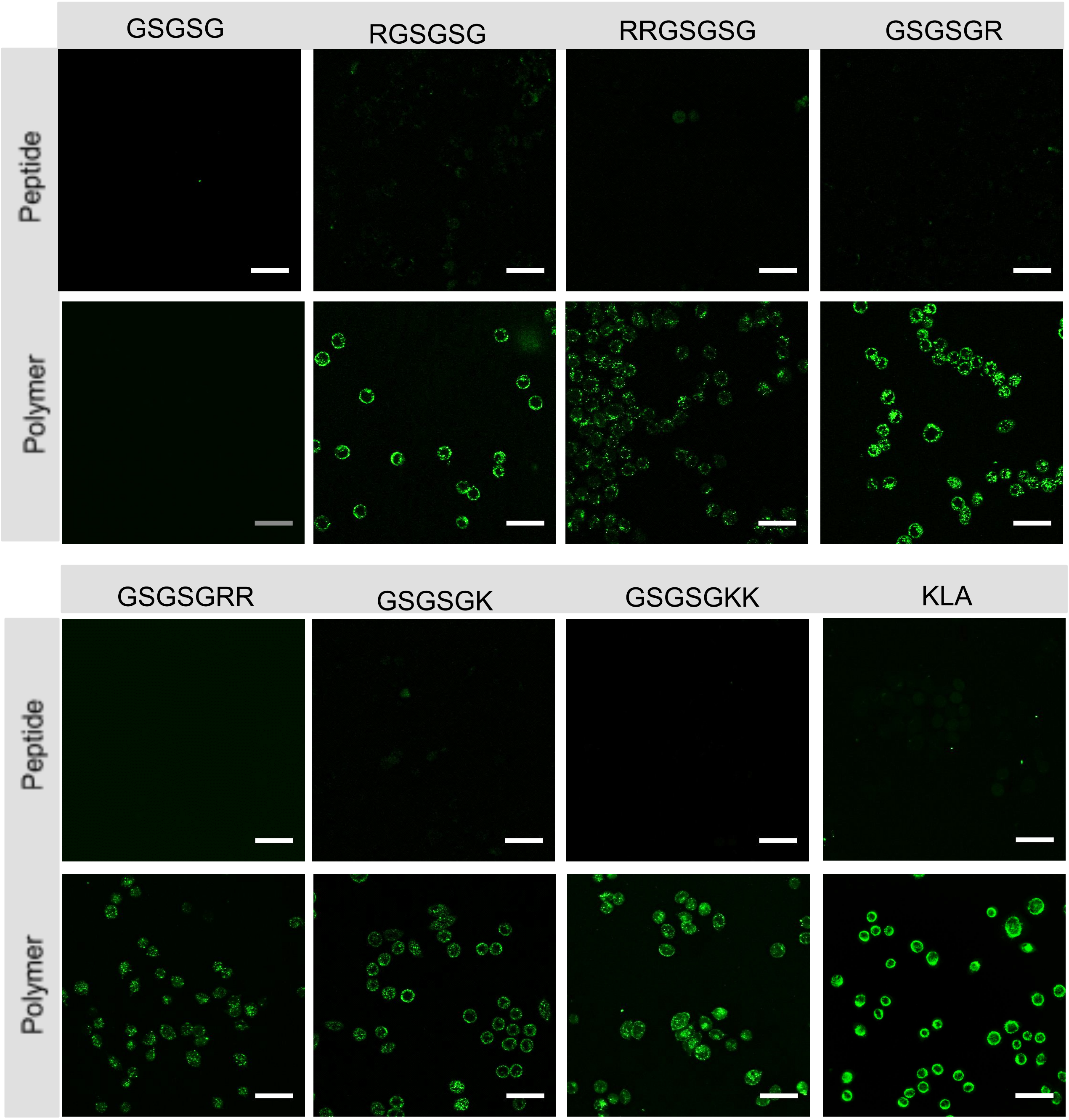

Figure S12. Live-cell confocal microscopy images of peptides and polymers. All images are the average intensity from six consecutive 1 μm Z-slices using a 40× objective. Cells were treated with 2.5 μM of the material (with respect to fluorophore). All GSGSG polymers and analogues have a peptide m of ~ 60 and the KLA polymer is m ~ 10. Scale bars are 50 μm. Each polymer that contains Lys or Arg shows a mixture of diffuse and punctate fluorescence, indicating that the material resides in the cytosol and in cellular compartments, respectively.

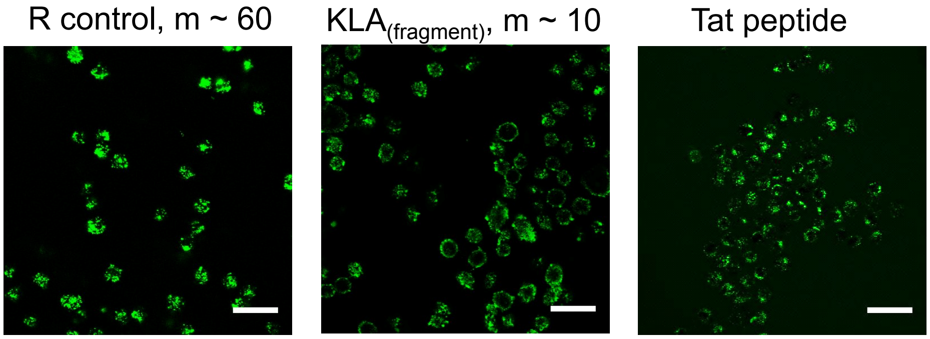

Figure S13. Live-cell confocal microscopy images of R control (DP or m of ~ 60), $KLA_{fragment}$ (m ~10) and Tat peptide. All images are the average intensity from six consecutive 1 μm Z-slices using a 40× objective. Scale bars are 50 μm. Each image shows a mixture of diffuse and punctate fluorescence, indicating that the material resides in the cytosol and in cellular compartments, respectively.

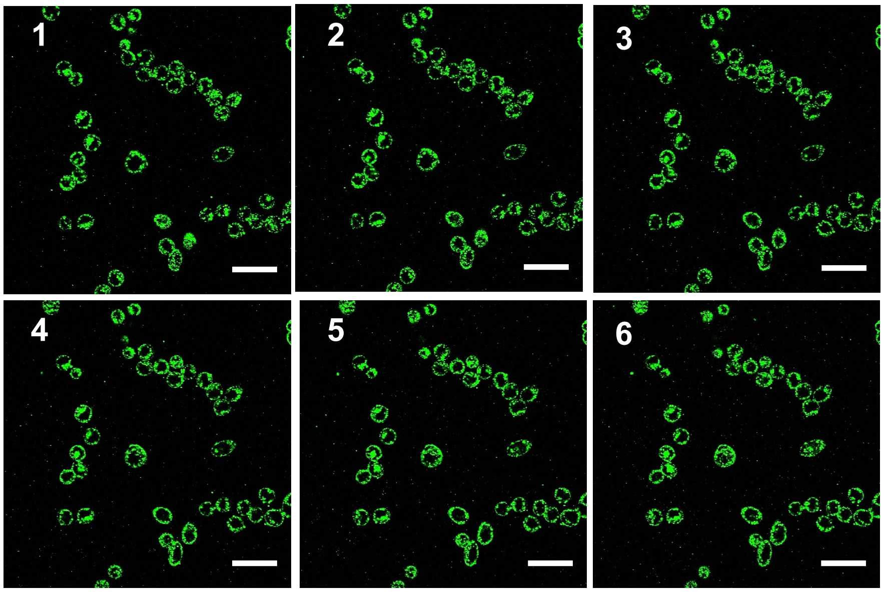

Figure S14. The six consecutive Z-slices that were averaged to yield the image for the GSGSGR polymer seen in Figure S12 and Figure 1C of the main text. The distance between each slice is 1 μm. Scale bars are 50 μm.

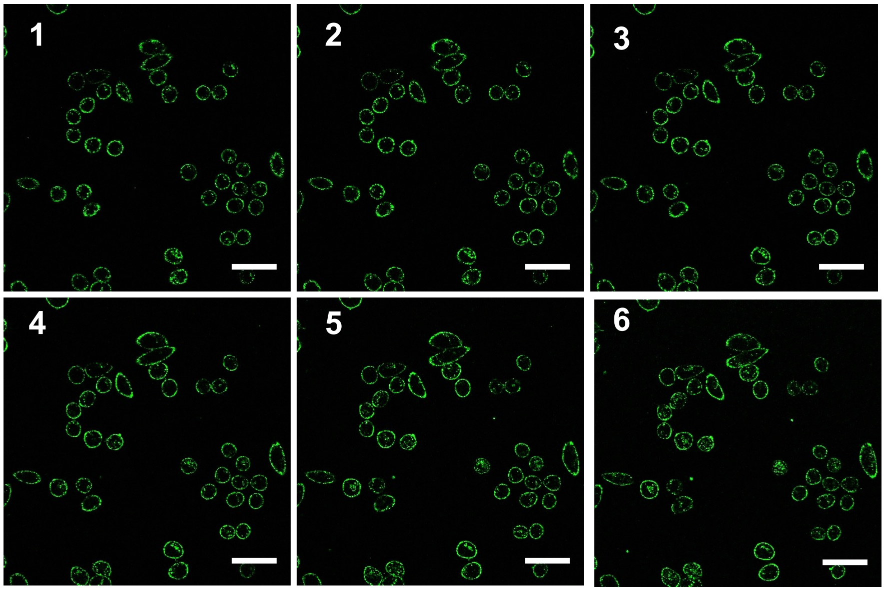

Figure S15. The six consecutive Z-slices that were averaged to yield the image for the GSGSGK polymer seen in Figure S12 and Figure 1C of the main text. The distance between each slice is 1 μm. Scale bars are 50 μm.

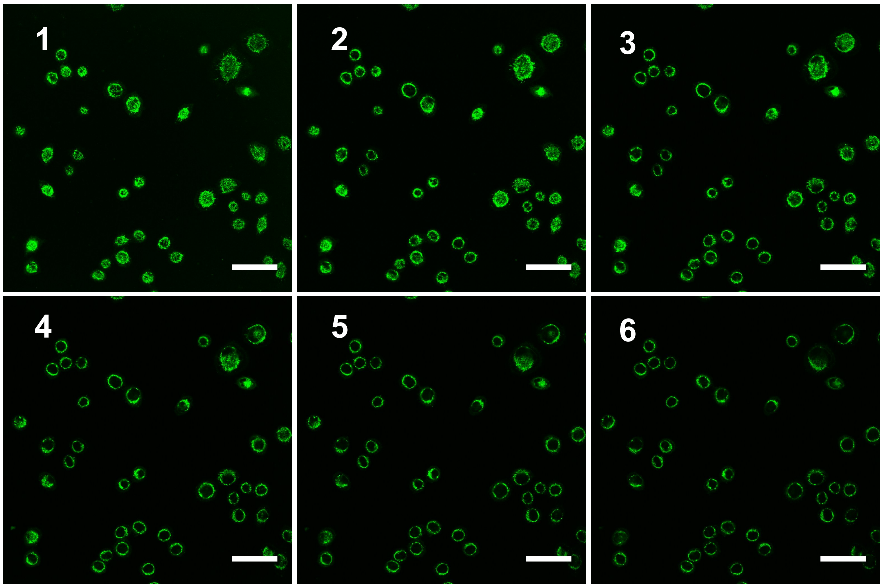

Figure S16. The six consecutive Z-slices that were averaged to yield the image for the KLA polymer seen in Figure S12 and Figure 2C of the main text. The distance between each slice is 1 µm. Scale bars are 50 µm.

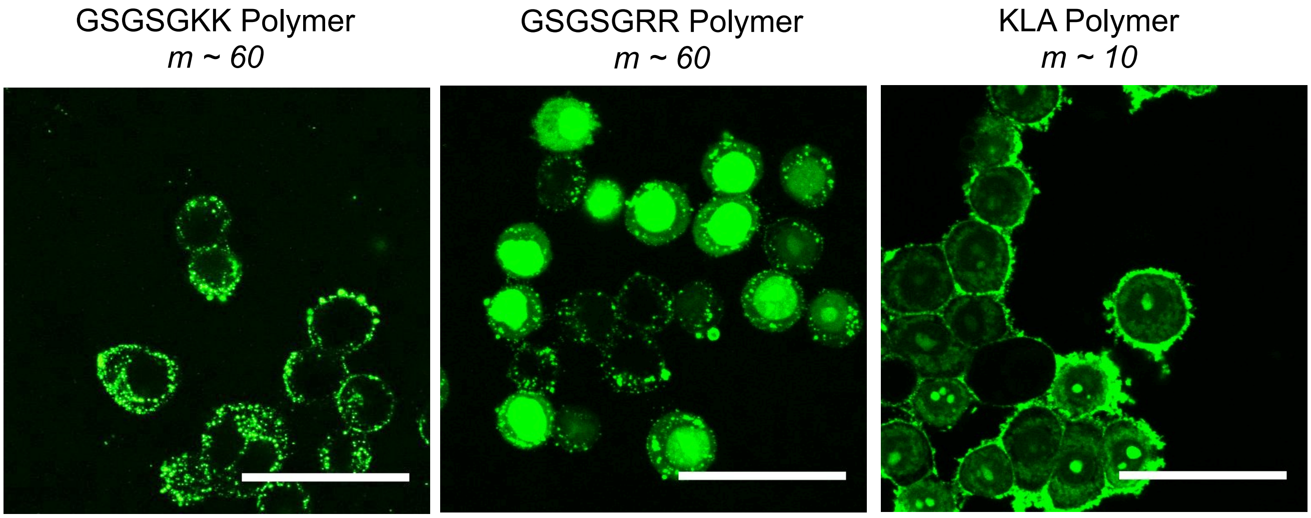

Figure S17. Larger resolution views of GSGSGRR, GSGSGKK and KLA Polymers. All images are the average intensity from six consecutive 1 µm Z-slices using a 100× objective. Cells were treated with 2.5 µM of the material (with respect to fluorophore). All GSGSG polymers and analogues have a peptide m of ~ 60 and the KLA polymer is m ~ 10. Scale bars are 50 µm. An assortment of diffuse and punctate fluorescence is present indicating that the material resides in the cytosol and in cellular compartments, respectively. This variability of localization is thus likely to be heavily dependent upon the identify of the peptide sequence and experimental conditions.

SVII. Cell viability assay. The cytotoxicity of materials was assessed using the CellTiter-Blue® assay (Promega, cat # G8081), which measures the metabolic reduction of resazurin to resorufin via fluorescence. For these studies, HeLa cells were plated at a density of 3,500 cells per well of a 96-well plate 18 hrs prior to treatment. Materials dissolved in DPBS at 10× the desired concentrations were added to the wells along with a 10% DMSO positive control. Cells were incubated for 72 hrs at 37 °C. Note that concentration for all toxicity measurements is with respect to peptide concentration to ensure that all peptides and polymers are fairly compared with respect to their therapeutic components. There is also no fluorophore present on the polymers or peptides used in these experiments to minimize potential background signals in the fluorescent measurements. The medium was removed and 80 µL of fresh medium lacking phenol red was added. To this was added 20 µL of the CellTiter-Blue® reagent and the cells were then incubated for 2 hrs prior to measuring fluorescence using 560 nm excitation and 590 nm emission. The fluorescence measurements were corrected for background fluorescence from the CellTiter-Blue® reagent by subtracting the fluorescence reading of wells treated with the reagent in the

absence of cells. Fluorescence values were then referenced as a percentage of the value obtained for the DPBS vehicle control.

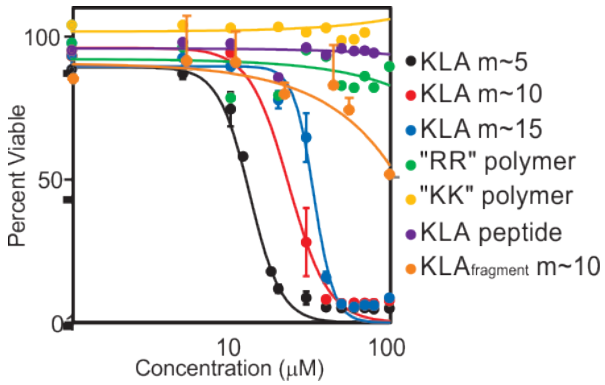

Figure S18. Comparison of cytotoxicity curves for KLA materials and controls. This figure is identical to that of Figure 2D in the main text, except it also contains the dose-response curves for the KLA (full length) polymers at m ~ 10 and 15.

SVIII. Circular dichroism. UV-Vis circular dichroism (CD) was used to evaluate whether the secondary structure of KLA, a mixture of random coil and alpha helix, is maintained upon polymerization. The peptide and polymer were dissolved in DPBS to a final concentration of 100 μM (with respect to peptide concentration). CD spectra were measured using an Aviv 215 spectrometer and each sample was measured from 190 to 260 nm with a slit width of 1 nm, scanning at 1 nm intervals with a 1s integration time. Measurements were taken 3× at 25°C and then averaged to give the spectra in Figure S19.

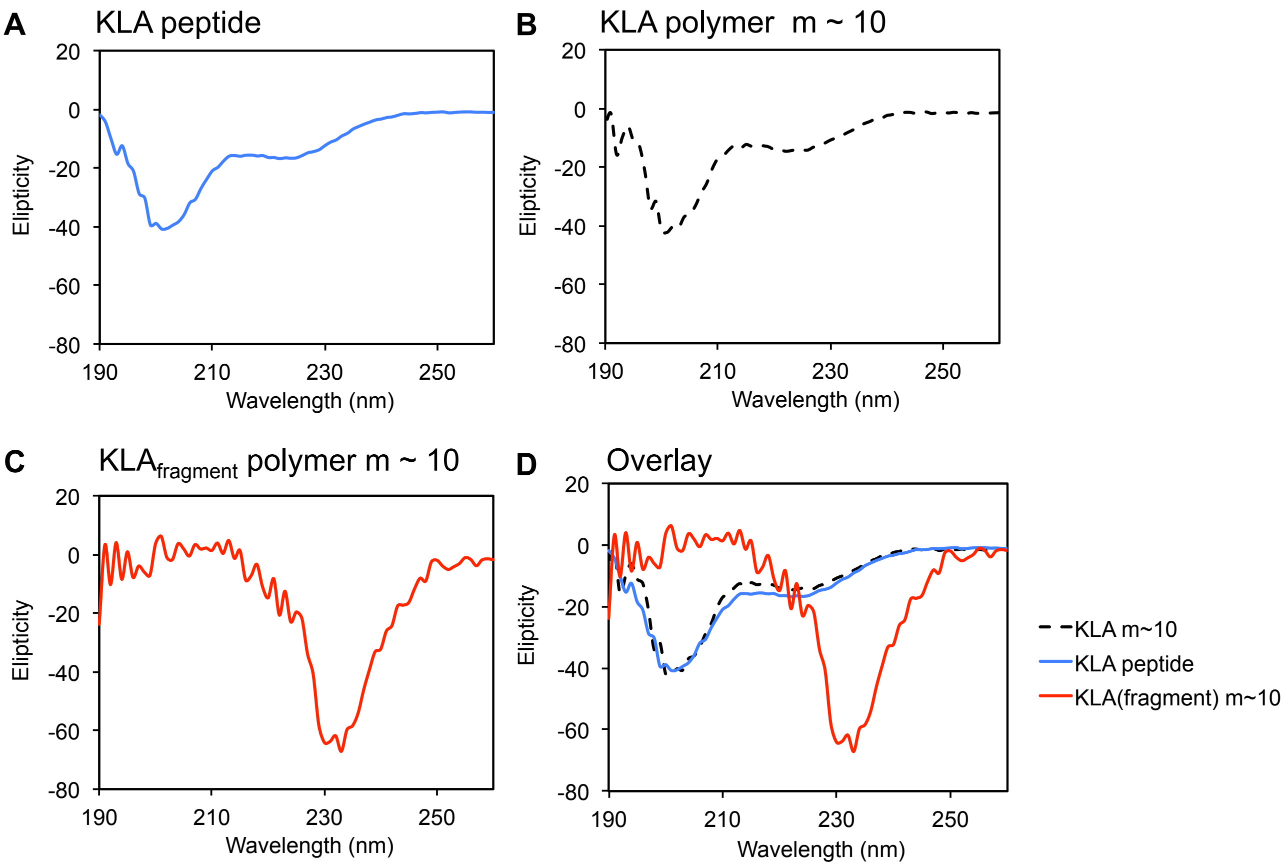

Figure S19. UV-Vis circular dichroism of KLA peptide and homopolymers. Spectra of (A) the KLA peptide, (B) the KLA homopolymer with m ~ 10, (C) the KLA$_{fragment}$ polymer with m ~10, and (D) an overlay of A, B and C. Spectra for the full length KLA peptide and polymer are nearly identical, indicating that polymerization of the KLA sequence does not perturb the secondary structure of the peptide. The spectrum of the KLA fragment polymer is different from the full-length constructs, suggesting that the secondary structure for this material is unique.

SIX. JC-1 Mitochondrial integrity assay. The mitochondrial membrane potential was measured using the MitoProbe® JC-1 assay kit (Life Technologies, cat # M34152), which measures the membrane potential using the JC-1 dye. The JC-1 dye forms J aggregates in healthy mitochondria that are red fluorescent, but cannot form the J aggregates when the mitochondria are disrupted, leading to a decrease of red fluorescence and an increase of green fluorescent J monomers in the cytosol. For these studies, HeLa cells were plated at a density of 90,000 cells per well of a 24-well plate 18 hrs prior to treatment. Materials dissolved in DPBS at 10× the desired concentrations (where concentration is with respect to peptide and no fluorophore is present) were added to the wells and incubated for 30 minutes. The materials were removed and the cells were washed with DPBS, then medium was added to the cells followed by incubation with 10 μL of a 200 μM solution of JC-1 dye to give a final concentration of 2 μM. To one set of wells, 2 μL of 50 mM carbonyl cyanide 3-chlorophenylhydrazone (CCCP) was added to give a final concentration of 50 μM. The small molecule CCCP is used as a positive control because it can associate with and depolarize mitochondrial membranes. The cells were incubated for 30 more minutes. The medium was then removed and the cells were washed 2× with DPBS and then incubated 3× for five minutes each with heparin (0.5 mg/mL in DPBS; Affymetrix, cat. # 16920), and finally rinsed again with DPBS. The cells were then trypsinized (0.25 % trypsin in DPBS; Gibco Life Tech., cat. # 15090-046) for 10 min, cold medium was added, and the cells were transferred to Eppendorfs. The suspended cells were then centrifuged to pellets and then resuspended in a minimal amount of cold DPBS. Flow cytometry data (10,000 events on three separate cultures per condition) was then acquired.

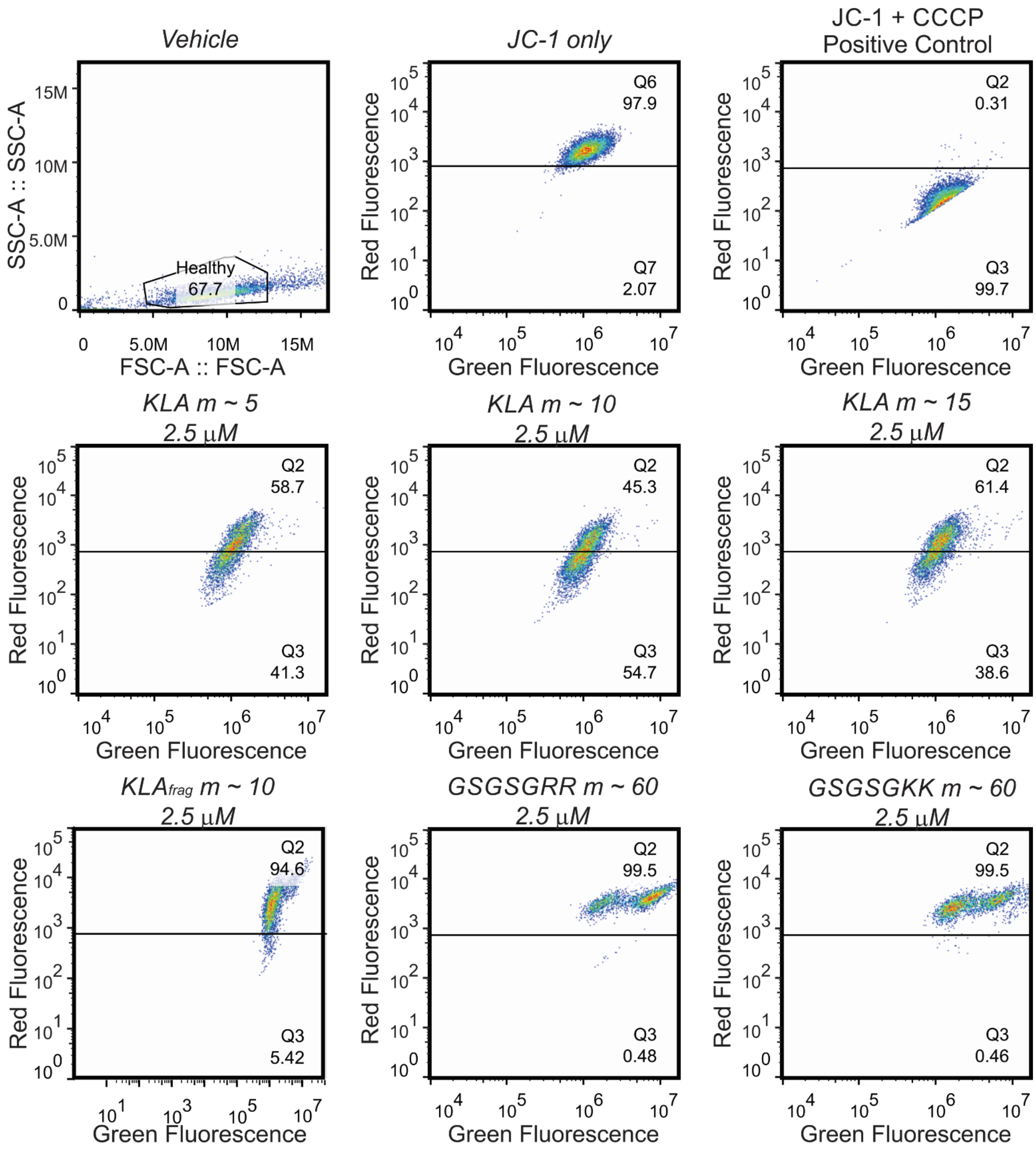

Figure S20. JC-1 assay for mitochondrial integrity. The samples were run on flow cytometry and each sample was gated using the vehicle control (DPBS). The quadrants were chosen based on where cells treated with CCCP (Q3) and cells that were untreated reside (Q2), with the line drawn between these two regions. Q2 is the percent of cells with healthy mitochondria, and Q3 is the percent of cells with the unhealthy mitochondria.

SX. Apoptosis and necrosis assays. Apoptotic and necrotic cells were identified using the CF™488A Annexin V and PI Apoptosis Kit (Biotium, cat # 30061), in which Annexin V (green) binds to apoptotic cells and PI (red) stains necrotic cells. For these studies, HeLa cells were plated at a density of 420,000 cells per well of a 6-well plate 18 hrs prior to treatment. KLA, m ~ 10, was dissolved in DPBS to a concentration of 2000 μM, where concentration is with respect to peptide and no fluorophore is present. Staurosporin, a small molecule that is known to cause apoptosis, was dissolved at 1000× (desired concentration on cells is 1 μM) in DMSO.

Staurosporin and the vehicle control were added to the wells 24 hrs prior to analysis, and KLA (m ~ 10) and 10% DMSO control were added 5 hr prior to analysis. After incubation, materials were removed and the cells were washed 2× with DPBS followed by 3 × 5 minute incubations with heparin (0.5 mg/mL in DPBS), and finally rinsed once with DPBS. The cells were then trypsinized (0.25% trypsin in DPBS) for 10 min, cold medium was added and the cells were transferred to Eppendorf tubes. The suspended cells were centrifuged to pellets and then resuspended in 100 μL Annexin V binding buffer. Then 5 μL of Annexin V was added to each Eppendorf, followed by 1 μL of PI. The cells were then incubated in a 37 ° C water bath for 15 minutes in the dark. After incubation, flow cytometry data was acquired monitoring the FITC channel (for Annexin V staining) and the PI channel.

SXI. Apoptosis marker assay. The expression of caspase enzymes, markers of apoptotic cells, in cells treated with KLA materials was assessed using the Apo-One® assay (Promega, cat # G7790), which measures the expression of caspase 3/7,using a fluorogenic substrate for those enzymes. For these studies, HeLa cells were plated at a density of 5,000 cells per well of a 96-well plate 18 hrs prior to treatment. Materials dissolved in DPBS at 10× the desired concentration (10 μM, where concentration is with respect to peptide and no fluorophore is present) were added to the wells. Cells were incubated for 30 minutes at 37 °C. The medium was removed the cells were washed 3× with DPBS. Then 100 μL of the Apo-One® reagent was added to the cells and to wells that contain no cells to get a baseline reading. The plate was mixed for 30 seconds using a shaker followed by incubation at 37 °C. After 3 hrs of incubation, the fluorescence was measured using an excitation of 499 nm and emission at 521 nm. The baseline fluorescence was subtracted from the fluorescence values for the wells, and fluorescence values were then referenced as a percentage of the value obtained for the DPBS vehicle control.

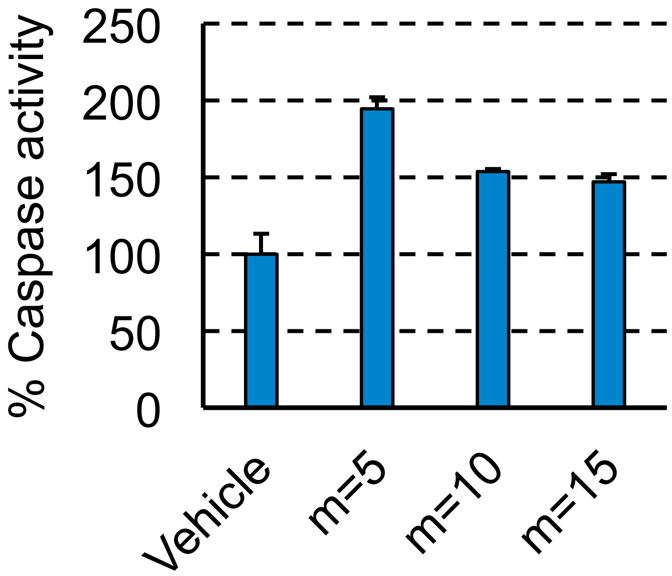

Figure S21. Levels of Caspase 3/7 in vehicle-treated cells and cells treated with KLA polymers at varying DP. The baseline level of expression is taken as 100% in the vehicle- treated cells. Those treated with polymers show an increase in expression, which is indicative of apoptosis. Each measurement was performed 3× on at least two separate subcultures.

SXII. Mechanistic studies by flow cytometry. For mechanistic studies, cells were plated and treated as described in the previous flow cytometry experiments, except here cells were preincubated with the indicated compound for 30 minutes at 37 °C prior to addition of the cell-penetrating material. The following concentrations were used: 80 μM dynasore (Enzo Life Sciences, cat. # 270-502-M005) and 9.5 mM MβCD (Fischer Scientific, cat # AC377110050). For studies at reduced temperature, cells were incubated at 4 °C for 30 min prior to and during incubation with the compound of interest. Note that an incubation duration of 30 m was chosen because no toxicity was observed during this time interval by any of the inhibitors, yet a clear and obvious impact on cellular uptake could be seen. For the reduced temperature studies only, all subsequent washes and manipulations were also done with ice-cooled media and other materials. Data is reported as the normalized mean fluorescence, which is the mean fluorescence yielded by the material divided by the mean fluorescence from the vehicle control. Each measurement was preformed 3× on at least three separate subcultures.

SXIII. RP-HPLC analysis of proteolytic susceptibility. The percent of intact GSGSGKK, GSGSGRR or KLA peptides and polymers after incubation with trypsin (Gibco Life Tech., cat. # 15090-046) or Pronase (Roche, cat. # 10165921001) was assessed by comparing RP-HPLC chromatograms. In these experiments, each peptide or

polymer (50 μM, where concentration is with respect to peptide content to ensure fair comparison between the peptides and polymers) was incubated with each protease (at 1 μM) for 3 hrs. At this point, the proteases were heat denatured at 65 °C for 15 m and the resulting solution was immediately injected onto an analytical RP-HPLC. Given that treatment with each protease often yielded multiple peptide fragments, a standard curve for each starting material (rather than every potential product) was prepared (Figure S22) to assess the percentage of intact material remaining after proteolytic digestion as given in Figure 4B of the main text. Note that the standard curves for polymers will be biased because the polymer backbone, OEG coblock and fluorophore should remain in tact after cleavage, and will therefore comprise part of the measured peak area. Nevertheless, no new peaks were seen in the chromatograms of any polymer post enzyme treatment (Figures S23-25), suggesting that these materials are not susceptible to cleavage by the proteases. Major fragments of the peptide controls were also identified by ESI MS. Consistent with the notion that the polymers are protected from proteolysis, no discernable peptide fragments were identified by MS in the polymer reaction mixtures.

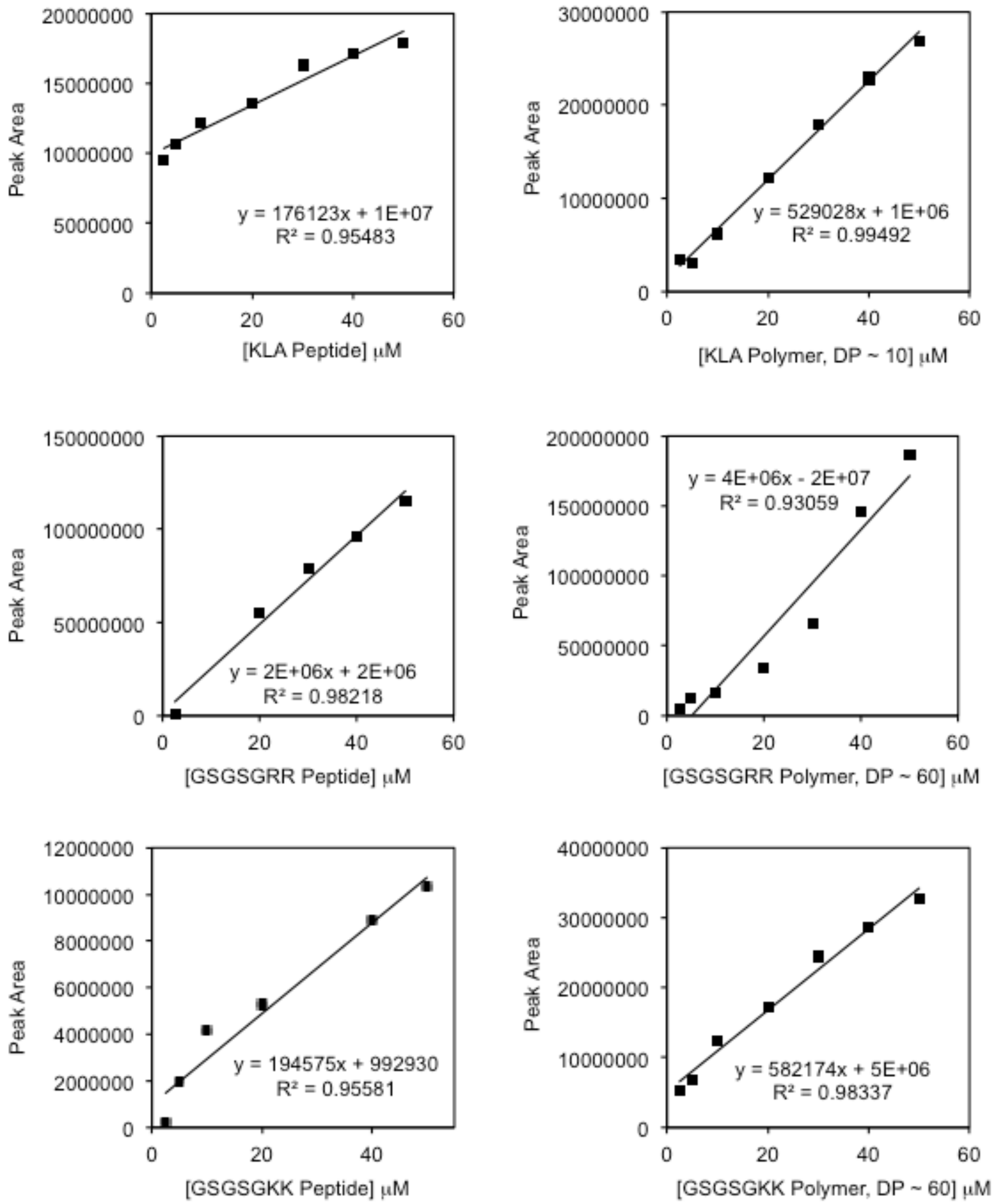

Figure S22. Standard curves, correlating peak area on RP-HPLC chromatograms with concentration on an 18 μL injection, for the determination of the concentration of intact peptide or polymer remaining after proteolytic cleavage. These standard curves were used to determine the extent of proteolytic cleavage for the chromatograms shown in Figures S23-24. Percent proteolytic cleavage for each material and condition is then given in Figure 4B in the main text. In all cases, concentration is with respect to the peptide content of each material.

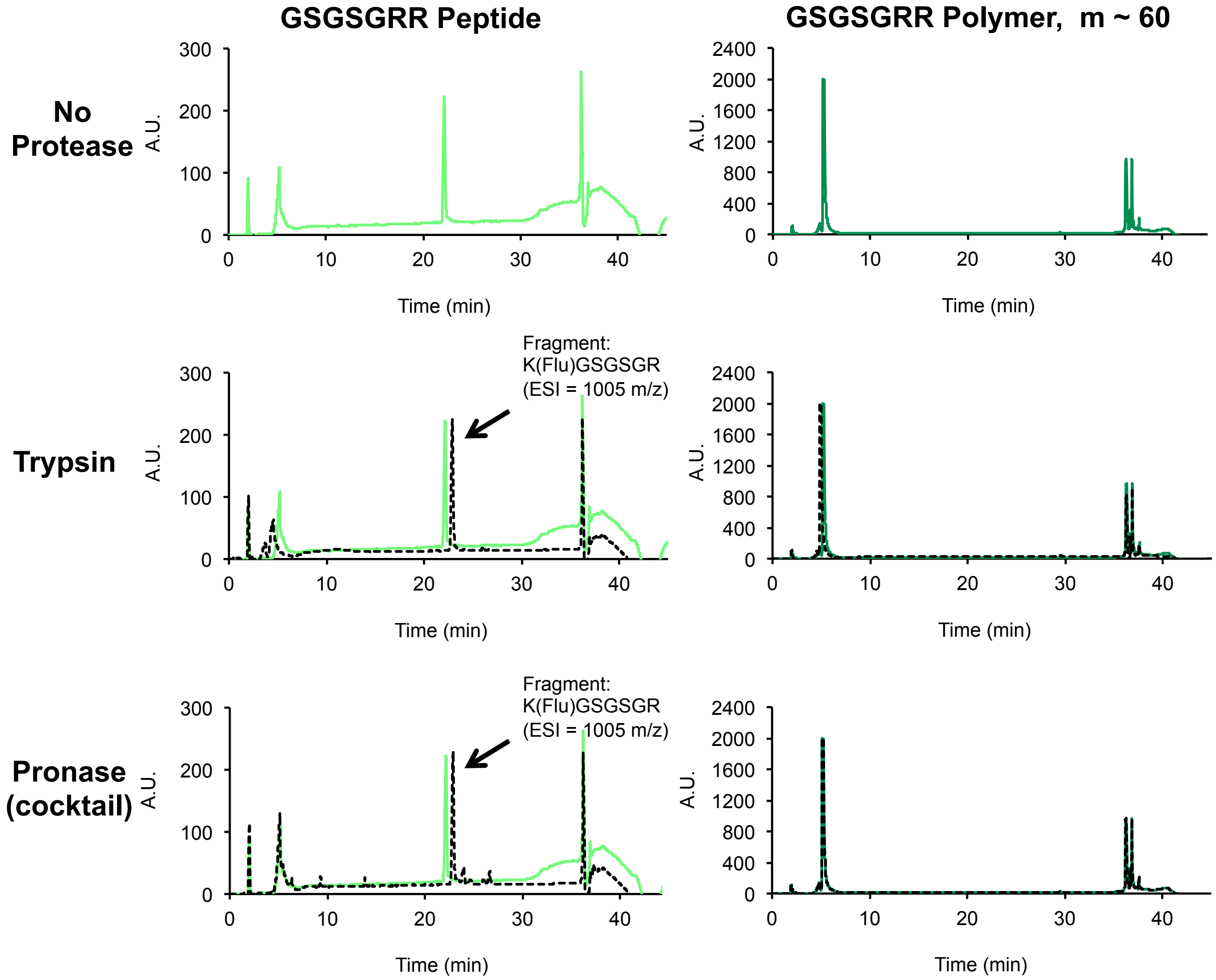

Figure S23. RP-HPLC assay of the proteolytic cleavage of K(Flu)GSGSGRR peptide and GSGSGRR polymer (m ~ 60) by pronase and trypsin. Each peptide/polymer (50 µM, with respect to peptide concentration) was incubated with the protease (1 µM) for 3 hr in DPBS. The trace in green is of the intact peptide or polymer that has not been treated with a protease and the dotted black trace is the same material after treatment with protease. The identity of the major peptide fragments were determined by ESI MS. These chromatograms were used, in combination with the standard curves given in Figure S22, to determine the extent of proteolytic cleavage given as provided Figure 4B of the main text.

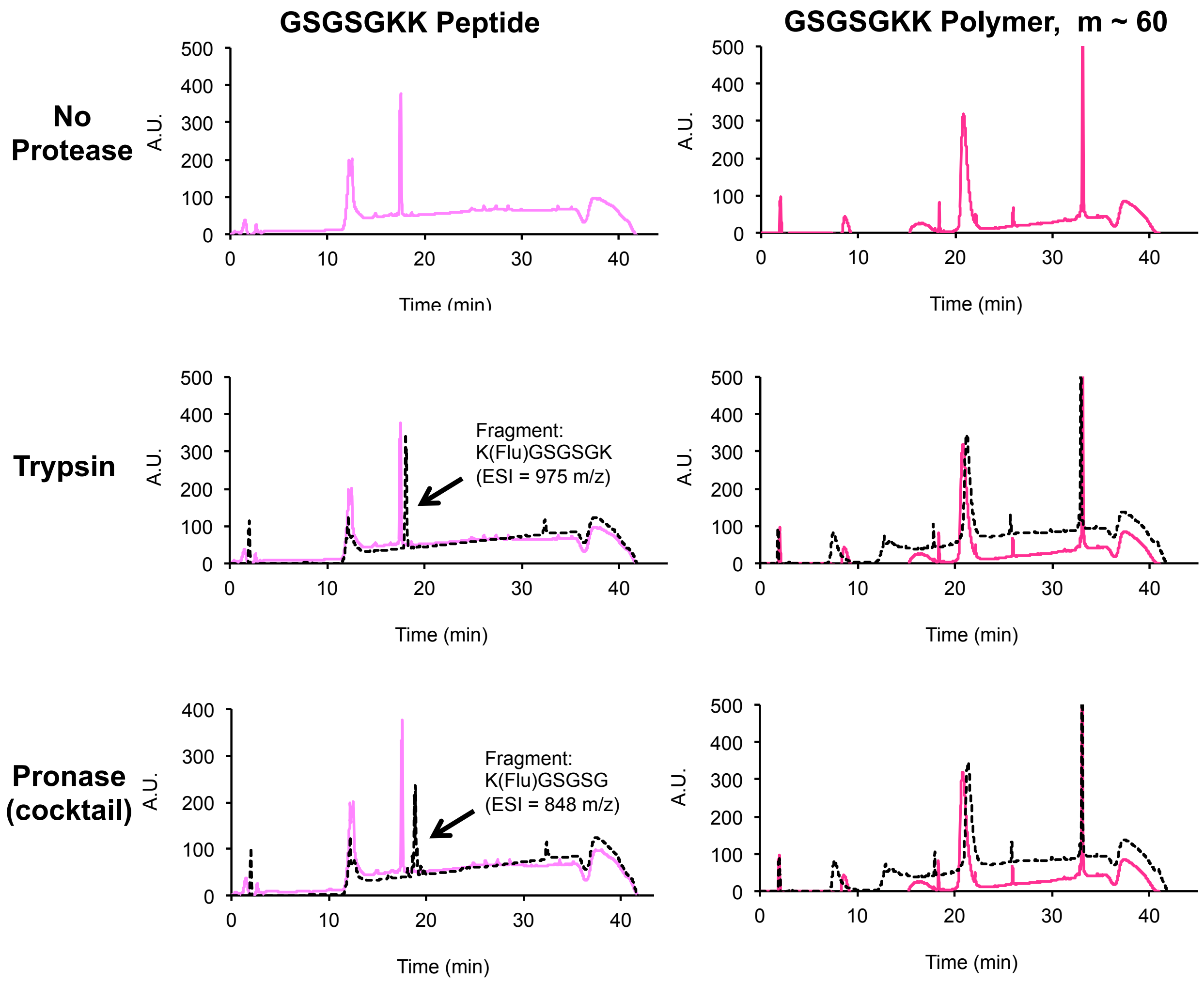

Figure S24. RP-HPLC assay of the proteolytic cleavage of K(Flu)GSGSGKK peptide and GSGSGKK polymer (m ~ 60) by pronase and trypsin. Each peptide/polymer (50 μM, with respect to peptide concentration) was incubated with the protease (1 μM) for 3 hr in DPBS. The trace in pink/purple is of the intact peptide or polymer that has not been treated with a protease and the dotted black trace is the same material after treatment with protease. The identity of the major peptide fragments were determined by ESI MS. These chromatograms were used, in combination with the standard curves given in Figure S22, to determine the extent of proteolytic cleavage as provided in Figure 4B of the main text.

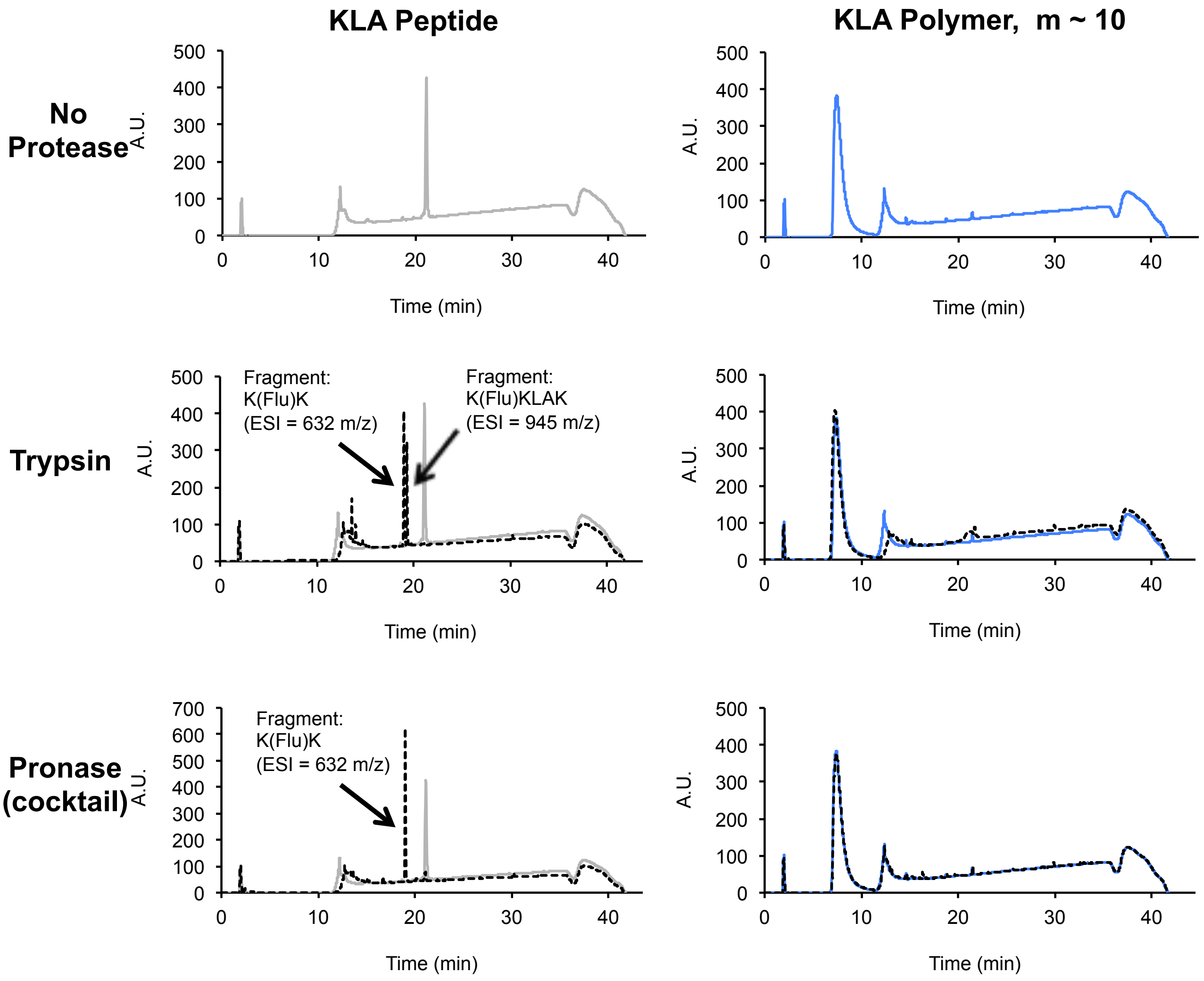

Figure S25. RP-HPLC assay of the proteolytic cleavage of K(Flu)KLAKLAKKLAKLAK peptide and KLAKLAKKLAKLAK polymer (m ~ 60) by pronase and trypsin. Each peptide/polymer (50 μM, with respect to peptide concentration) was incubated with the protease (10 μM) for 3 hr in DPBS. The traces in grey and blue are of the intact peptide or polymer that has not been treated with a protease and the dotted black trace is the same material after treatment with protease. The identity of the major peptide fragments were determined by ESI MS. These chromatograms were used, in combination with the standard curves given in Figure S22, to determine the extent of proteolytic cleavage as provided in Figure 4B of the main text.

REFERENCES

[1] A. P. Blum, J. K. Kammeyer, J. Yin, D. T. Crystal, A. M. Rush, M. K. Gilson, N. C. Gianneschi, *J. Am. Chem. Soc.* **2014**, *136*, 15422-15437.
[2] M. P. Thompson, L. M. Randolph, C. R. James, A. N. Davalos, M. E. Hahn, N. C. Gianneschi, *Polym. Chem.* **2014**, *5*, 1954-1964.
[3] M. S. Sanford, J. A. Love, R. H. Grubbs, *Organometallics* **2001**, *20*, 5314-5318.
[4] P. R. Patel, R. C. Kiser, Y. Y. Lu, E. Fong, W. C. Ho, D. A. Tirrell, R. H. Grubbs, *Biomacromolecules* **2012**, *13*, 2546-2553.
[5] R. M. Conrad, R. H. Grubbs, *Angew. Chem.* **2009**, *48*, 8328-8330.
[6] I. M. Kaplan, J. S. Wadia, S. F. Dowdy, *J. Controlled Release* **2005**, *102*, 247-253.
